# Quantitatively relating magnetic resonance *T*_1_ and *T*_2_ to glycosaminoglycan and collagen concentrations mediated by penetrated contrast agents and biomacromolecule-bound water

**DOI:** 10.1093/rb/rbad035

**Published:** 2023-04-11

**Authors:** Jingming Gao, Xian Xu, Xiaoye Yu, Ye Fu, Hongjie Zhang, Siyi Gu, Dinglingge Cao, Quanyi Guo, Liming Xu, Jiandong Ding

**Affiliations:** State Key Laboratory of Molecular Engineering of Polymers, Department of Macromolecular Science, Fudan University, Shanghai 200438, China; Department of Radiology, The Second Medical Center & National Clinical Research Center for Geriatric Diseases, Chinese PLA General Hospital, Beijing 100853, China; State Key Laboratory of Molecular Engineering of Polymers, Department of Macromolecular Science, Fudan University, Shanghai 200438, China; State Key Laboratory of Molecular Engineering of Polymers, Department of Macromolecular Science, Fudan University, Shanghai 200438, China; State Key Laboratory of Molecular Engineering of Polymers, Department of Macromolecular Science, Fudan University, Shanghai 200438, China; State Key Laboratory of Molecular Engineering of Polymers, Department of Macromolecular Science, Fudan University, Shanghai 200438, China; State Key Laboratory of Molecular Engineering of Polymers, Department of Macromolecular Science, Fudan University, Shanghai 200438, China; Institute of Orthopedics, The First Medical Center, Chinese PLA General Hospital, Beijing Key Lab of Regenerative Medicine in Orthopedics, Key Laboratory of Musculoskeletal Trauma and War Injuries of PLA, Beijing 100853, China; Institute for Medical Device Control, National Institutes for Food and Drug Control, Beijing 102629, China; State Key Laboratory of Molecular Engineering of Polymers, Department of Macromolecular Science, Fudan University, Shanghai 200438, China

**Keywords:** magnetic resonance imaging, collagen, glycosaminoglycan, bound water, cartilage

## Abstract

Magnetic resonance imaging (MRI) is a promising non-invasive method to assess cartilage regeneration based on the quantitative relationship between MRI features and concentrations of the major components in the extracellular matrix (ECM). To this end, *in vitro* experiments are performed to investigate the relationship and reveal the underlying mechanism. A series of collagen (COL) and glycosaminoglycan (GAG) solutions at different concentrations are prepared, and *T*_1_ and *T*_2_ relaxation times are measured with or without a contrast agent (Gd-DTPA^2−^) by MRI. Fourier transform infrared spectrometry is also used to measure the contents of biomacromolecule-bound water and other water, allowing theoretical derivation of the relationship between biomacromolecules and the resulting *T*_2_ values. It has been revealed that the MRI signal in the biomacromolecule aqueous systems is mainly influenced by the protons in hydrogens of biomacromolecule-bound water, which we divide into inner-bound water and outer-bound water. We have also found that COL results in higher sensitivity of bound water than GAG in *T*_2_ mapping. Owing to the charge effect, GAG regulates the penetration of the contrast agent during dialysis and has a more significant effect on *T*_1_ values than COL. Considering that COL and GAG are the most abundant biomacromolecules in the cartilage, this study is particularly useful for the real-time MRI-guided assessment of cartilage regeneration. A clinical case is reported as an *in vivo* demonstration, which is consistent with our *in vitro* results. The established quantitative relation plays a critical academic role in establishing an international standard ISO/TS24560-1:2022 ‘Clinical evaluation of regenerative knee articular cartilage using delayed gadolinium-enhanced MRI of cartilage (dGEMRIC) and *T*_2_ mapping’ drafted by us and approved by International Standard Organization.

## Graphical abstract

**Figure:**
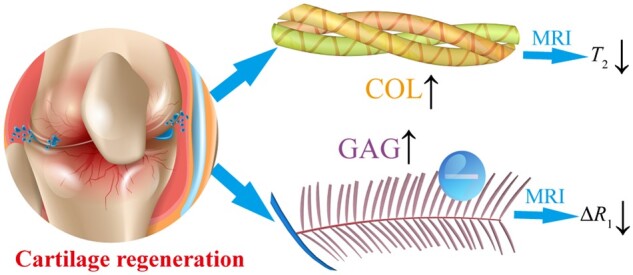

## Introduction

Various injuries, inflammation or degeneration may cause irreversible damage to articular cartilage [1–5]. It is important to make an accurate diagnosis and treatment of articular cartilage injury. The basic quantitative index comes from the assessment of cartilage thickness and degree of joint smoothness, etc., leading to some relevant scoring systems such as Mankin, Outerbridge, Bentley or Ficat scoring systems to evaluate cartilage status based on arthroscopic observations and biopsies [6–9]. However, most of the approaches that characterize tissue regeneration lead to injury and are thus not ideal for clinical assessment. Magnetic resonance imaging (MRI) can perform a non-invasive evaluation of the entire joint, with obvious contrast between the lesion area and the adjacent normal cartilage tissue area [10]. Compared with computed tomography and other imaging methods, MRI has a higher contrast of soft tissue and is more suitable for the evaluation of articular cartilage.

**Figure 1.**
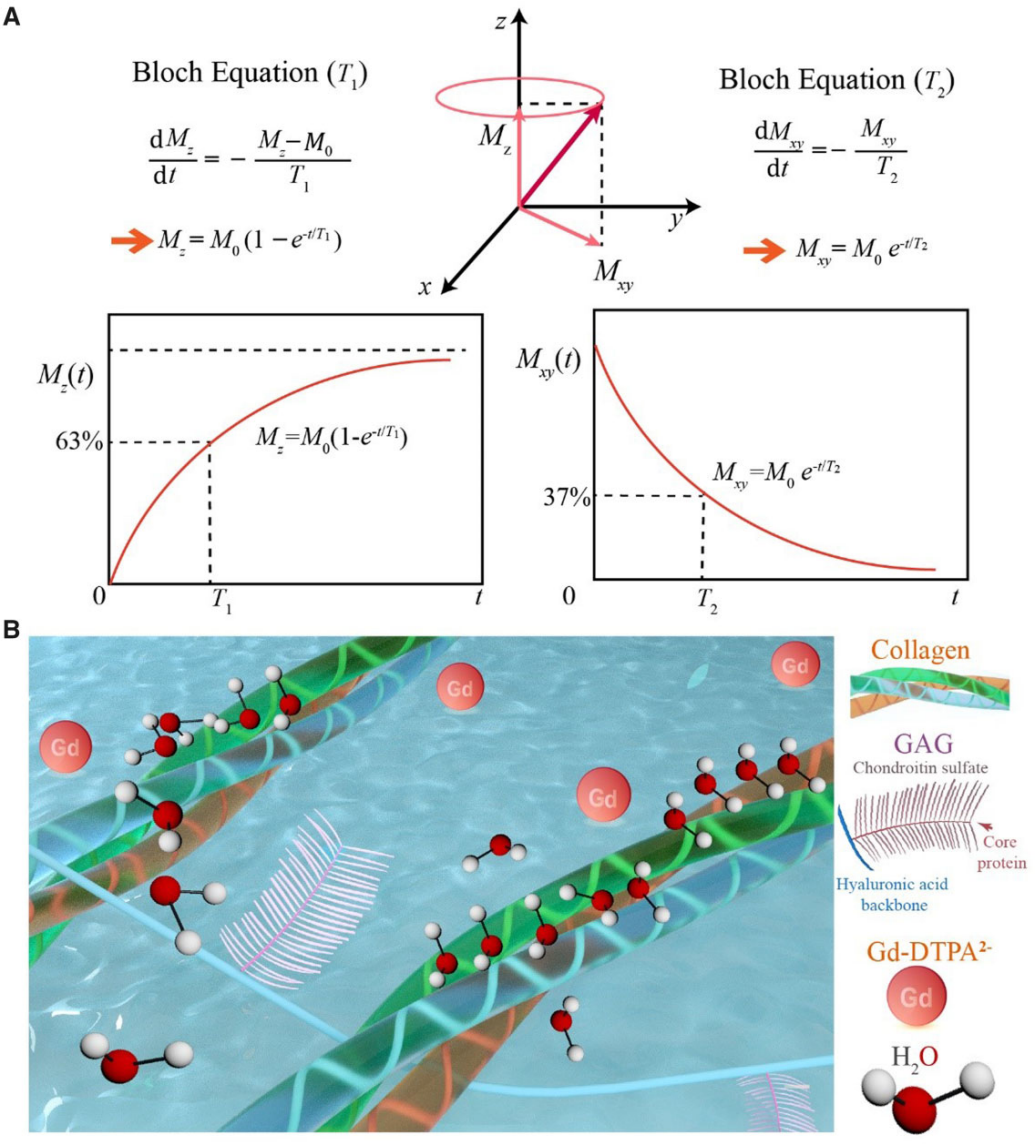
Schematic diagram of magnetic resonance *T*_1_ and *T*_2_ and the main components in cartilage ECM affecting these two characteristic quantities. (**A**) The NMR phenomenon appears when a system of nuclei in a static magnetic field experiences an RF. After the RF pulse is removed, longitudinal magnetization (*M_z_*) is restored owing to spin–lattice relaxation, and transversal magnetization (*M_xy_*) is decayed owing to spin–spin relaxation. Demonstrated in the figure is the relaxation after removal of a 90° RF. Here, *M*_0_ denotes the modulus of the magnetic vector under the static magnetic field prior to the RF. (**B**) The magnetic resonance signal comes from the relaxation of the hydrogen protons of the water molecules in the cartilage, while the relaxation of the water molecules is affected by the surrounding COL and GAG and the applied contrast agent.

The MRI signals relevant to this study come from protons in the hydrogen nuclei. The magnetic moment produced by the spin of the proton tends to be parallel to the external static magnetic field ***B*_0_**, which is usually defined as the *z* direction, forming the longitudinal magnetization vector ***M*_0_**. After being excited by the radiofrequency (RF) pulse at the Larmor frequency, the protons resonate and thus deflect the longitudinal magnetization vector from the *z*-axis to the *xy*-plane. After the RF pulse ceases to excite, the protons rearrange along the ***B*_0_** magnetic field and release excess energy, and the electromagnetic signal in this process constitutes that of nuclear magnetic resonance (NMR). Bloch *et al.* [11] proposed two characteristic magnetic resonance (MR) times *T*_1_ and *T*_2_ and put forward the Bloch equation, as schematically shown by us in Fig. 1A, demonstrated by the relaxation after a 90° RF pulse. The *T*_1_ relaxation refers to the recovery process of the longitudinal magnetization vector along the axis of the static magnetic field, and the *T*_2_ relaxation refers to the decay process of the transverse magnetization vector.

Articular cartilage is composed of extracellular matrix (ECM) and chondrocytes. Whether inside or outside the cells, water is the most important component. In addition to water molecules, the two most abundant components in cartilage ECM are collagen (COL) and glycosaminoglycans (GAG), as shown in Fig. 1B. The schematic diagram of the triple helix structure of COL is shown in Supplementary Fig. S1. *T*_2_ imaging and *T*_1_-related delayed gadolinium-enhanced MRI imaging of cartilage (dGEMRIC) are employed to access cartilage regeneration. In the normal articular cartilage, ECM contains mainly type II COL (15–22%), proteoglycans (4–7%) and water (60–80%). During cartilage regeneration progress, the COL content in the cartilage tissue gradually increases, corresponding to an increase in bound water content and a decrease in free water (FW) proportion, ultimately leading to a decrease in *T*_2_. Meanwhile, the GAG content in the cartilage gradually recovers with the regeneration of cartilage tissue. Owing to the negative charge characteristics of GAG, the corresponding tissue charge density increases, while the relative permeability of gadolinium contrast agents with the same negative charge properties decreases, ultimately leading to a decrease in Δ*R*_1_.

While some excellent studies about MRI of cartilage have been reported both *in vivo* and *ex vivo* [12–17], it is interesting that the *in vitro* experiments in a well-defined ‘clean’ system of COL, GAG and their mixture without other dry components in cartilage are rare. According to our trial, it is not easy to adjust the viscosities of the examined biomacromolecular aqueous solutions to match the clinical MRI apparatus with appropriate pulse sequences and parameters. The theme of this research is to establish the relationship between COL and GAG contents to MR indexes *T*_1_ and *T*_2_ and interpret the underlying principle, mainly based on *in vitro* experiments.

## Experimental

### Liquid NMR analysis of COL

The NMR signals of COL aqueous solutions (99.9 wt% deuterated water) were acquired in a liquid NMR spectrometer (Bruker, AVANCE III HD, 400 MHz). COL solutions of 2, 5 and 10 wt% were prepared in heavy water in this work. The program of the excitation pulse sequence for this test was ZG, the 90° pulse width was 9.19 μs, the interval between repeated pulses was 6 s and the number of scans was 16.

### *T*_2_ test of biomacromolecule samples

Triple helix COL (type II) and GAG were from Yuanye Bio-Technology Co., Shanghai. In the pre-experiment of *T*_2_ mapping of COL solutions, we found that the *T*_2_ values were very large because of the large water content and good fluidity of water. To better mimic the viscosity of the ECM within the cartilage tissue, we introduced 10 wt% of poly(vinyl alcohol) (PVA, 2488) to increase the initial sample viscosity. COL solutions at different concentrations (0%, 3%, 6%, 9%, and 12%) with or without PVA were prepared.

The viscosity was measured with a rotational rheometer (Malvern, KinexusPro) equipped with a 1° cone-plate 60 mm in diameter. The gap between the upper and lower plates was chosen at 0.03 mm. The volume of the macromolecular solution was about 1.5 ml, and the excess sample was scraped off after the upper plate was closed. The samples were placed between the cone-plate and the platform, with a layer of silicone oil applied to the outer ring of the plate to avoid evaporation of water. The measurement method was chosen oscillating shear mode. The shear strain was set at 1% and the angular velocity was 10 rad/s. The dynamic rheological test was performed at 25°C with data points taken every 2 s. For each group of samples, the average value of 100 points was taken as the sample viscosity.

**Figure 2.**
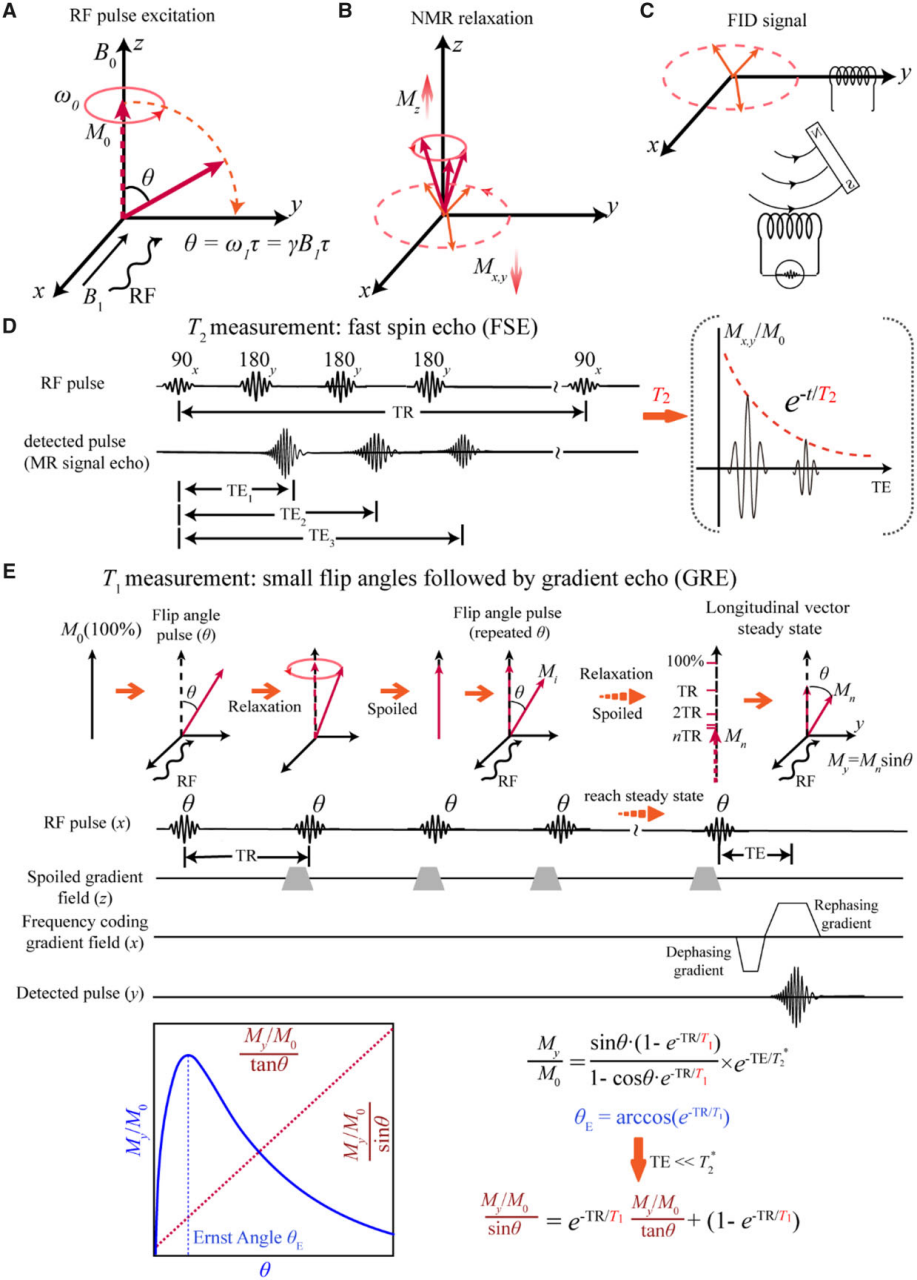
Schematic presentation of the principle and method of *T*_2_ and *T*_1_ measurements in MRI. (**A**) Diagram of the RF pulse excitation process. The RF pulse excites the sample from the *x*-axis, making the hydrogen proton precess around the *z*-axis and deflect to the *y*-axis at the same time. (**B**) Relaxation of the proton occurs after the removal of RF, including longitudinal spin–lattice relaxation and transverse spin–spin relaxation. (**C**) The FID signal is obtained on the *y*-axis coil by the relaxation electromagnetic wave. (**D**) A 90° pulse at Larmor precession frequency is applied, followed by the 180° pulses to create an echo, and the sequence is repeated. The process of the exponential decay of the transverse magnetization vector is recorded. (**E**) The flip angle *θ* is the rotation angle of the excitation pulse consisting of 2–4 different small angles. The smaller rotation angle enables rapid recovery of relaxation, which leads to faster imaging.

*T*_2_ mapping was tested with a 3.0 T system (Skyra, Siemens) and a 20-channel coil. We summarize the test parameters in Table 1. Specifically, a fast spin echo sequence was adopted to perform multiple echo time (TE) tests in a long repetition time (TR). The schematic representation of MR detection and pulse sequence is shown in Fig. 2. TE indicates the time between the first pulse and the MRI echo signal, and TR means the time of the repetition of two RF pulses. The schematic diagram of the functions of 90° and 180° pulses is shown in Supplementary Fig. S2.

**Table 1.** Parameters of MR examination *in vitro* and in *vivo*

	*In vitro*	*In vivo* (human)
Equipment	3.0 T system (Skyra, Siemens, Germany); 20-channel coil	3.0 T system (Skyra, Siemens, Germany); 15-channel phased-array knee coil
*T*_1_-weighted mapping using a gradient echo and multi-flip angles	TR: 15 ms, TE 2.35 ms; flip angle: 5°, 12°, 19°, 26°	TR: 15 ms, TE: 2.7 ms; flip angle: 5, 26
*T*_2_-weighted mapping using multi-echo *T*_2_-weighted sequence	TR: 4030 ms; TE: 50, 100, 150, 200; 250, 300, 350, 400, 450, 500, 550, 600, 650, 700, 750, 800, 850, 900, 950 and 1000 ms	TR: 1921 ms; TE: 13.8, 27.6, 41.4, 55.2 and 69.0 ms

### *T*_1_ tests of biomacromolecule samples

Different concentrations of COL and GAG aqueous solutions were configured and tested for the effect of these two biomarker macromolecules of cartilage on *T*_1_ values. *T*_1_ mapping was carried out with a 3.0 T system (Skyra, Siemens) and a 20-channel coil. The sequence and principle of *T*_1_ value calculation are shown in Fig. 2E, while test parameters are listed in Table 1. Our *T*_1_ measurements were carried out using a gradient echo (GRE) sequence based on multi-flip angles. The complete schematic diagram of the GRE MR sequence and the schematic diagram of the classical *T*_1_ test are shown in Supplementary Figs. S3 and S4, respectively.

To simulate the process of the clinical intravenous injection of a contrast agent into the body, we set up a dialysis device to mimic the influence of different concentrations of biomacromolecules on the penetration of the contrast agent. Dialysis tubes with different concentrations of GAG and COL solutions were prepared, and the gadolinium contrast agent Gd-DTPA^2−^ (Magnevist, Schering) was added with the initial concentrations of 200, 400 or 1000 µmol/l. The corresponding *T*_1_ values and the difference in relaxation rate Δ*R*_1_ were calculated before and after the addition of the gadolinium contrast agent. With a 3.0 T system (Skyra, Siemens) and a 20-channel coil, *T*_1_ mapping was performed both before and after dialysis of gadolinium contrast agent with the following parameters: TR/TE 15/2.35 ms. For the detection of the concentration of gadolinium contrast agent in solution, we employed UV–visible spectrum scanning (Lambda, Perkin-Elmer) and inductively coupled plasma atomic emission spectrometer (ICP-AES, Thermo Fisher Scientific).

### Spectroscopic analysis of biomacromolecule samples

COL and GAG with different water contents were configured to analyze the location of the characteristic peaks and the proportion of bound water in them. In our spectroscopic characterizations, we first vacuum-dried COL or GAG. Then, 100, 200, 400 and 1000 μl of water were added to 0.5 g of dried biomacromolecules. The infrared spectra were measured with an infrared spectrophotometer (Nicolet 6700; Thermo Fisher) equipped with an attenuated total reflectance attachment with the diamond as the crystal material. All spectra were acquired after the background acquisition, and the final spectra minus the background signal were collected. The corresponding split-peak fitting according to the Gaussian peak was performed with Origin (OriginLab). In addition, we carried out Raman spectroscopy (XploRA, JobinYvon) of these biomolecule samples with different water contents. The relatively viscous samples were placed on glass slides and those with good flowability were placed in capillary tubes for testing. The spectroscopic measurements were conducted at room temperature and tested as quickly as possible to prevent water evaporation.

### Matrix-induced autologous chondrocyte transplantation

The clinical trial scheme was approved by the Medical Ethics Committee of the PLA General Hospital. Cartilage tissue engineering was performed to regenerate articular cartilage after matrix-induced autologous chondrocyte transplantation (MACI). As usual, MACI was carried out with natural or synthetic materials as scaffolds and with autologous chondrocytes as seed cells. In this study, we first carried out an arthroscopic assessment of the injury and obtained normal cartilage from the non-weight-bearing area of the knee joint. The tissue after aseptic packaging was sent to a laboratory that meets the Good Laboratory Practice standard. After digestion, the cells were cultured and enlarged for 4 weeks. The collected cells were loaded into scaffolds. The details of the methodology have been published previously [18–20]. After 24 h, the ECM-derived scaffolds and seed cells were transplanted into the injured area by a second surgery to regenerate cartilage.

### MRI observations of clinical cartilage regeneration

MRI was performed with a Siemens Skyra system (3.0 T) for patients at 3, 6 and 12 months after MACI treatment. A 15-channel phased-array knee coil was used, and the parameters are listed in Table 1. The experiments were performed periodically with repeated parameters to ensure the stability of the MRI tests. Prior to imaging, the patient should rest for >30 min to avoid any mechanical load that could affect the knee test parameters.

### Imaging analysis

To compare the regenerated cartilage with the healthy cartilage, the normal cartilage region in the same anatomical area was selected as the control, which was defined as the normal signal. The region of interest (ROI) of regenerated cartilage was manually drawn by experienced musculoskeletal radiologists, and its location was determined by at least two radiologists to ensure the accuracy of ROI localization. For imaging analysis, the ROI of regenerated cartilage should cover the thickness of the entire cartilage. We measured *T*_2_ and *T*_1_ values. In the latter case, *T*_1pre_ and *T*_1post_ with respect to the longitudinal relaxation times before and after the injection of the contrast agent were recorded, and Δ*R*_1_ (1/*T*_1post_ − 1/*T*_1pre_) was used to evaluate the GAG content in cartilage.

### Statistical analysis

All the data are shown as mean ± standard deviation and treated by one-way analysis of variance. It is considered to have a significant difference between the two groups if the corresponding *P*-values is <0.05. The data are demonstrated as ‘*’ for 0.01 < *P *<* *0.05, ‘**’ for 0.001 < *P *<* *0.01 and ‘***’ for *P *<* *0.001.

## Results

### Analysis of COL solutions in a conventional liquid NMR apparatus

**Figure 3.**
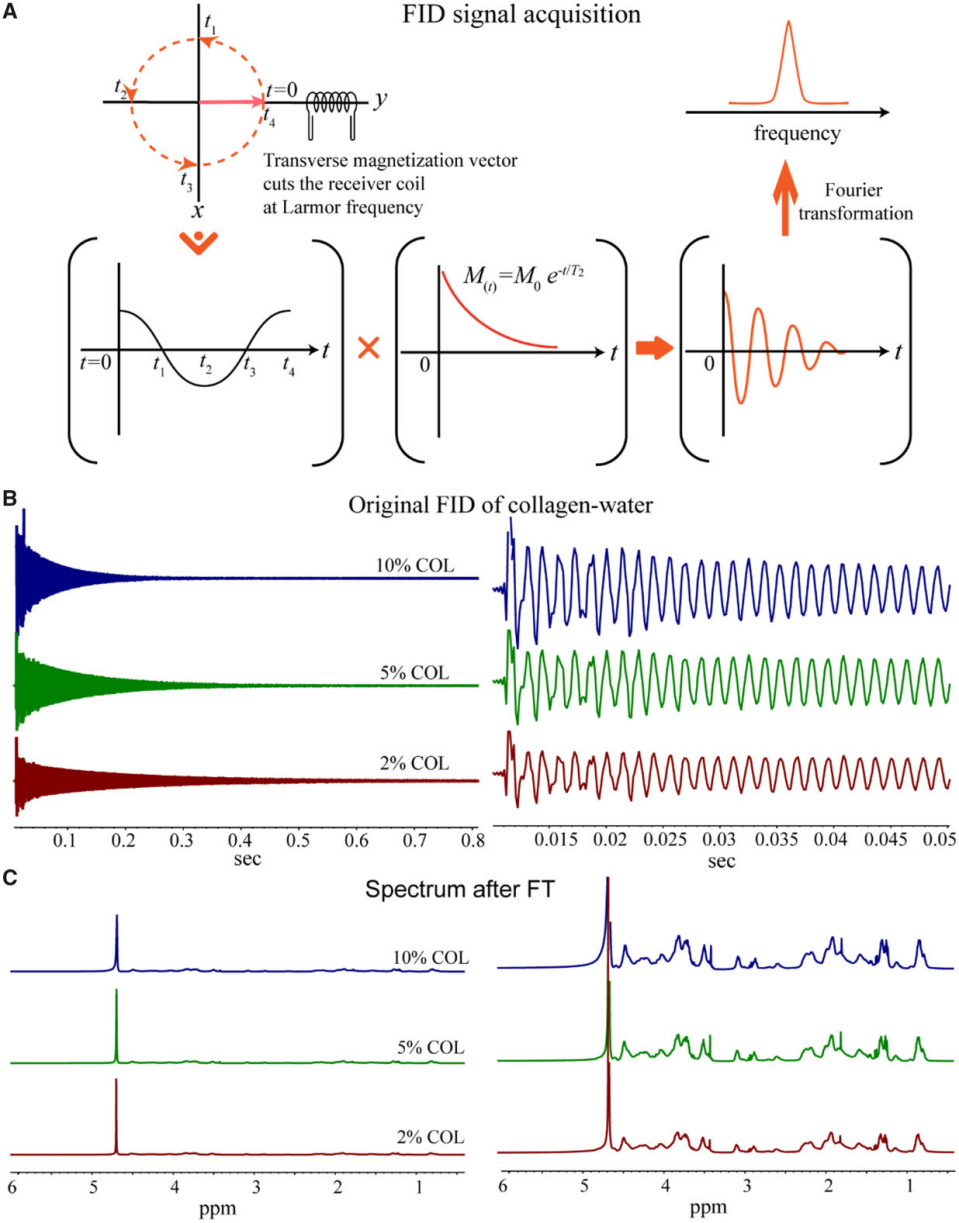
NMR analysis of COL aqueous solutions. (**A**) Schematic diagram of the FID signal, which would be transformed from a time domain signal to a frequency domain signal after the Fourier transform. (**B**) Typical initial FID signals of the COL aqueous solutions of the indicated three concentrations. The right shows the FID signals within short record time. (**C**) The spectra of the COL solutions after Fourier transform. The right shows the magnified signals of the left.

We first detected the H NMR of COL solutions in 99.9 wt% deuterated water. The free induction decay (FID) signal and the chemical shift diagram after Fourier transform (FT) are shown in Fig. 3. The significant NMR signals of the COL solutions appeared at 4.8 ppm, which comes from H_2_O and is much sharper than the signal of the COL itself. Even with very low water content, the H_2_O signal in the NMR is still predominant over COL.

We estimated the proportion of H from different sources in COL samples as follows. Take the 10 wt% COL in 0.5 ml heavy water (99.9%) as an example. COL consists of different amino acids, and the average molecular weight of its amino acid units is 110 g/mol according to the abundance of different amino acids in the protein. So, 0.05 g of COL contains 0.45 mmol of amino acids. If each amino acid residue contains 5 hydrogen protons, the COL molecules contain 2.25 mmol hydrogen protons. The 0.5 ml of 99.9% heavy water solvent contains 0.0005 g of residual H_2_O water molecules, which corresponds to 0.03 mmol of water molecules including 0.06 mmol of hydrogen protons. In the 10 wt% COL in deuterated water, the number of hydrogen protons in COL is about 40 times of that in H_2_O, but the signal in hydrogen protons of H_2_O is much larger than that of the COL molecules. Hence, the NMR relaxation signal of the COL solution comes from the H_2_O signal. As shown in Fig. 3C, the solvent water peaks gradually widen with the increase of [COL], indicating that *T*_2_ became shorter. This conventional NMR detects in aqueous solutions of COL are consistent with the later results of *T*_2_ mapping in a clinical MRI apparatus.

### *T*_2_ values of *in vitro* biomolecule samples with different concentrations from a clinical MRI apparatus

**Figure 4.**
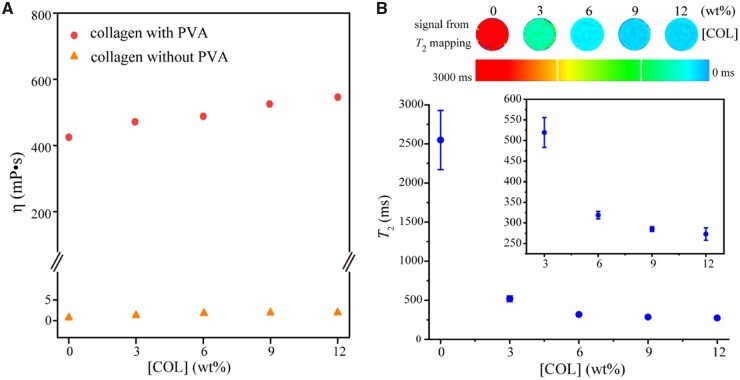
The viscosities of COL aqueous solutions and the relationship between *T*_2_ value and COL concentration. (**A**) The viscosity increased slightly with the COL concentration, after 10 wt% water-soluble synthetic polymer PVA was added to significantly increase the initial viscosity of the biomacromolecule-water system. (**B**) *T*_2_ values at different COL concentrations in presence of 10 wt% PVA. For each group, *n *=* *3.

All of the remaining MR data were from a clinical MRI apparatus. As shown in Fig. 4A, the addition of a synthetic polymer PVA (PVA-2488, 10 wt%) significantly increased the viscosity of a COL aqueous solution. In contrast, the viscosity η of the corresponding system in the presence of PVA increased slightly with the increase of COL concentration, from about 420 to 550 mP s in the examined range.

The *T*_2_ value decreased significantly with the increase of COL concentration, as shown in Fig. 4B. Considering that *T*_2_ reflects the spin–spin relaxation of the hydrogen protons in the water molecules in the system, the above results implied the binding of some water molecules to COL chains. The high COL concentration leads to the increase of the bound water content, which accelerates the dipole–dipole interaction of hydrogen protons and finally leads to the decrease of the *T*_2_ value. Supplementary Fig. S5 shows the effect of the GAG solution and the mixture of COL/GAG solution on *T*_2_ values. The *T*_2_ value decreases with the increase of GAG content, although the decreasing trend is not as significant as the increase of COL content.

### Spectroscopic analysis

To further analyze how different concentrations of biomacromolecules affect *T*_2_ value, we conducted spectroscopic analysis and made theoretical derivation of the possible quantitative relationship between biomacromolecule concentration and *T*_2_ value. It has been reported that infrared or Raman spectroscopy of vibrations of aqueous solutions has a wide spectral peak in the range of 3000–3700/cm [21, 22]. Symmetric stretching, asymmetric stretching and hydrogen bond vibration of water molecules are within this range (Supplementary Fig. S6). The different frequency locations reflect the influence of different neighboring structures on water molecules and hydrogen bonding. Researchers have carried out peak segmentation processing of 2–6 peaks for the signal at this place [23–27]. Nakamoto *et al.* [28] found that compared with ‘unbound water’, ‘bound water’ forms shorter hydrogen bonds and lower vibrational peaks of corresponding water molecules. Therefore, for the IR spectra of water systems, the difference between bound water and FW can be reflected from the different frequencies of O–H vibrational peaks. The infrared vibration modes of different chemical bonds are schematically presented in Supplementary Fig. S7.

**Figure 5.**
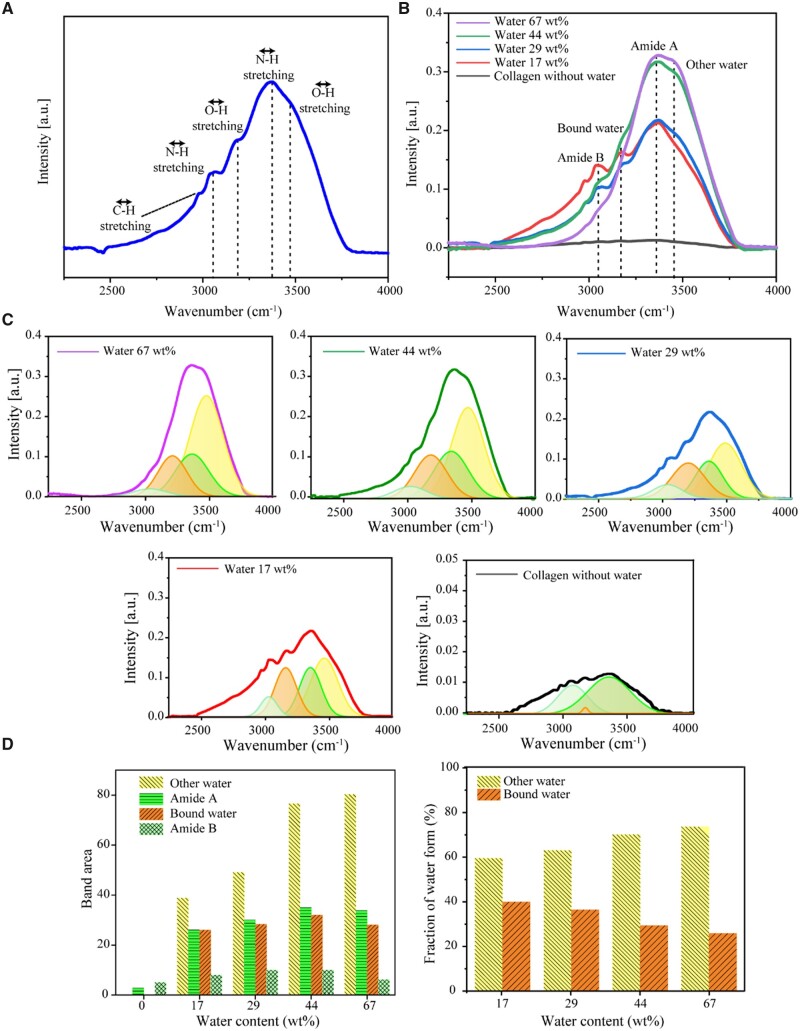
FTIR of COL-water systems with highly concentrated COL by dropping the indicated contents of water into dried collagen samples. (**A**) a typical FTIR spectrum with the absorption peaks identified as follows: OH stretching (other water) ∼3470/cm, NH stretching (Amide A) ∼3340/cm, OH stretching (bound water) ∼3200/cm, NH stretching (Amide B) ∼3070/cm and CH stretching ∼2950/cm. The formation of hydrogen bonds reduces the force constant of the chemical bond and the absorption frequency shifts to a low wavenumber. (**B**) A series of FTIR spectra of the COL-water system with various water contents. The samples were prepared by adding 100 µl, 200 µl, 400 µl and 1 ml of water to 0.5 g of dried COL. The IR peak positions and their assignments are indicated. (**C**) Spectrum fitting of OH and NH stretching bands by four Gaussian components. (**D**) Fitting areas of different stretching bands and different ratios of the two water forms, bound water and other water.

Referring to some literature [29, 30], we treated the infrared peaks of the COL-water system with four characteristic peaks, as shown in Fig. 5. Here, the stretching vibration of O–H is divided into the bound water peak (∼3200/cm) and the other water peak (∼3470/cm). The term ‘bound water’ refers to water molecules that are tightly bound to COL or other solutes, while the term ‘other water’ refers to other water molecules that are self-bonded to form a water network. The infrared peak position of the amide bond can be divided into nine locations, in which Amide A and Amide B are mainly in the characteristic region, and the other Amides I–VII are mainly near the fingerprint region [31]. Amide A and Amide B defined in this work are near 3300 and 3100/cm, which are in good agreement with the literature [29–31].

The positions of the IR characteristic peaks and the corresponding peak separation of the GAG-water system were also tested, with the results shown in Supplementary Fig. S8. Compared to COL, the proportion of bound water of GAG is significantly smaller than the bound water content of COL. This implies that COL influences the formation of bound water in cartilage significantly.

We also carried out Raman spectroscopy. The results for COL and GAG are shown in Supplementary Fig. S9. For the peak positions of the Raman signals of the biomacromolecule-water system, one can refer to Leikin *et al.* [32], where the C–H stretching vibration signals of biomacromolecules are at 2800–3050/cm while the O–H and N–H stretching vibration signals are at 3100–3700/cm. Since the viscous samples are placed on slides for direct testing while the solution samples are tested in capillaries, the comparison between the absolute values of the Raman signals does not make sense. The only source of the C–H signal is the biomolecules of the sample, which can be normalized according to the signal at C–H. Similar to the IR peak identification, the unbound water signal is at a higher frequency. The results show reasonably that the fraction of unbound water increases with the total water content.

### Theoretical derivation of the relationship between *T*_2_ values and COL concentrations and determination of the fraction of outer-bound water after fitting the experimental data combining both MR and IR measurements

We hypothesized that the bound water signal detected via IR spectroscopy is attributed to only the first layer of water molecules directly bound to biomolecules, which can be called inner-bound water (IBW). Because each water molecule tends to form four hydrogen bonds, this inner layer of bound water affects the orientation and mobility of several adjacent layers of water molecules, which we define as outer-bound water (OBW). Other water molecules have relatively large mobility, which is closer to the situation of water molecules without biomacromolecules and is simply called FW. Strictly speaking, the so-called FW here is also composed of hydrogen bonds with neighboring water molecules, but not a single water molecule. Obviously, OBW cannot exist without IBW.

We further hypothesized that the quantity of OBW is much greater than that of IBW and, thus, its influence on *T*_2_ is significant. Based on this hypothesis, the FID of MRI is derived from IBW protons with signal intensity *M*_IBW_, OBW protons with signal intensity *M*_OBW_ and FW proteins with signal intensity *M*_FW_, namely:


(1) $$M\;=\;M_{\mathrm{IBW}}\;+\;M_{\mathrm{OBW}}+\;M_{\mathrm{FW}}.$$

We denote the fraction of bound water as *f*_IBW_ and those of OBW and FW as *f*_OBW_ and *f*_FW_, respectively. Then, the reduced signals *M*_IBW_, *M*_OBW_ and *M*_FW_ are, respectively, decayed as


(2) $$M_{\mathrm{IBW}}=\;f_{\mathrm{IBW}}\;\exp(-t/\tau_{\mathrm{IBW}}),$$

(3) $$M_{\mathrm{OBW}}=\;f_{\mathrm{OBW}}\;\exp(-t/\tau_{\mathrm{OBW}}),$$

(4) $$M_{\mathrm{FW}}\;=\;f_{\mathrm{FW}}\;\exp(-t/\tau_{\mathrm{FW}}).$$

Here, τ_IBW_, τ_OBW_ and τ_FW_ are the *T*_2_ relaxation times of IBW, OBW and FW, respectively.

The relaxation time of the OBW is uncertain, because its motility might increase from the inside to the outside. For simplicity, we assume that the average *T*_2_ relaxation time of OBW is the geometric average of the relaxation time of IBW and FW. Based on the MRI results in Fig. 4B, it is reasonable for us to assume 2500 ms as τ_FW_, 250 ms as τ_OBW_ and 25 ms as τ_IBW_ in this study. A similar classification of MRI water signals into three different states of water is seen in some brain MR analyses [33, 34]. In these reports, the brain MR signal is reduced to that of water, which is divided into three cases: one is the long *T*_2_ component produced by cerebrospinal fluid (∼2 s); the second is the intermediate component of intracellular and extracellular water (∼70–100 ms); the third is the short *T*_2_ component (∼10–20 ms) of water trapped between the double layers of myelin.

In our opinion, the concentration of IBW depends on the accessible surface area of biomacromolecules. Theoretically, [IBW] is related to [COL] by a 2/3 power at a low concentration of COL. So, the IBW content *f*_IBW_ with respect to total water has the following relationship with its concentration [IBW] relative to the sum of water and COL as


(5) $$f_{\mathrm{IBW}}\;=\frac{\left[\mathrm{IBW}\right]}{1-[\mathrm{COL}]}\;∼\;\frac{{\left[\mathrm{COL}\right]}^{2/3}}{1-[\mathrm{COL}]}.$$

Here, [COL] is the mass percent concentration, and the equation is valid only when [COL] < 1.

With the increase of COL concentration [COL], the overlap of COL molecules must lead to a decrease in the ratio of bound water. So, the above formula needs to be multiplied by a parameter that decreases with the increase of [COL]. While the analytical form is unknown, we can speculate that if the final bound water fraction is a power of COL concentration, the power is certainly <2/3.

Based on the peak separation results of bound water and other water by IR in Fig. 5, the fractions of bound water and unbound water in the COL-water system with different concentrations can be obtained, and then, the relationship between the content of bound water and the concentration of COL can be deduced, supposing a boundary condition that the content of ‘COL-bound water’ in pure water be regarded as zero. Hence,


(6) $${f_{\mathrm{IBW}}\;=\;\alpha [\mathrm{COL}]}^{n}$$

**Figure 6.**
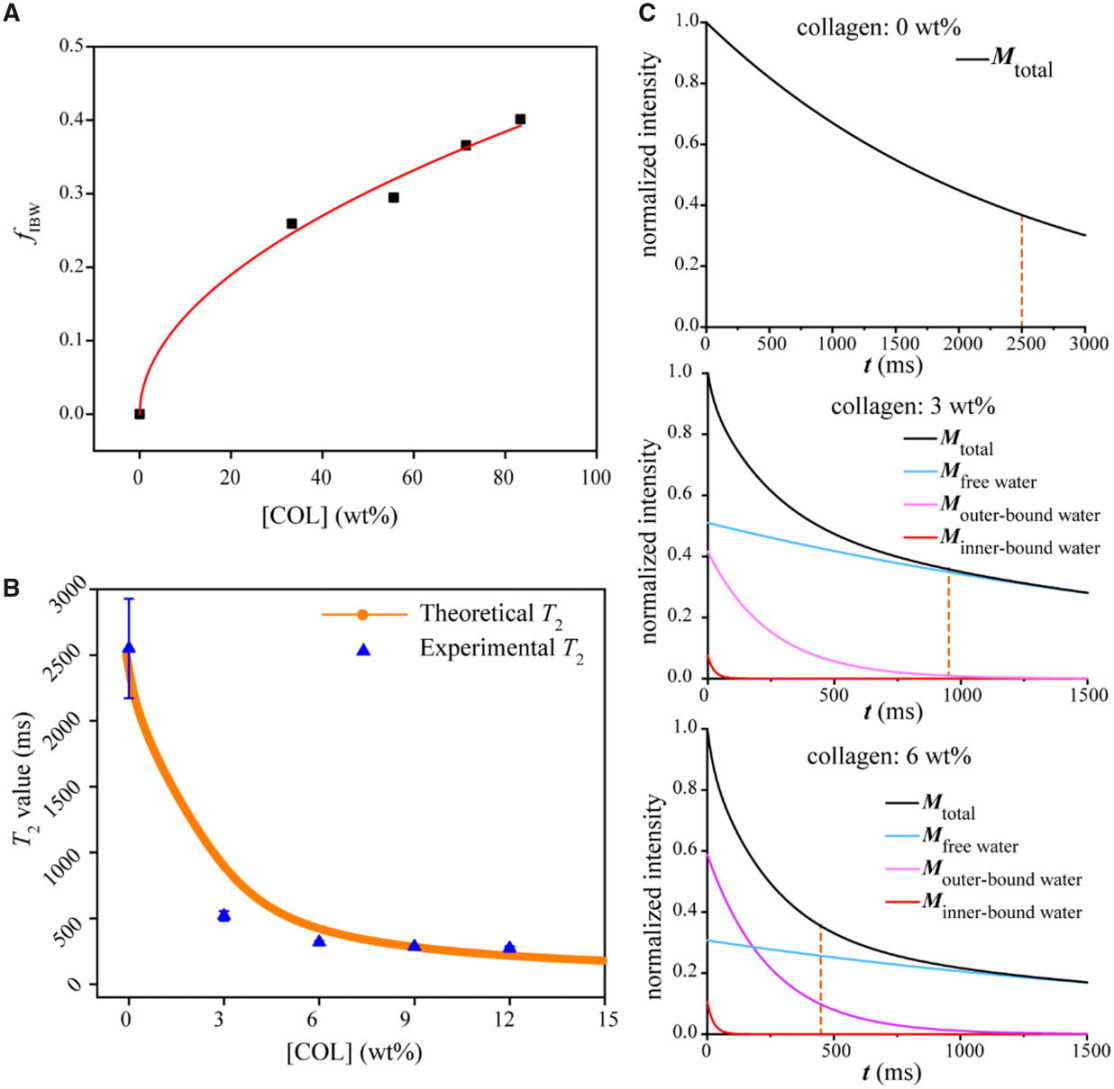
Theoretically derived MRI signals and comparison with experimental *T*_2_ values at different COL concentrations. (**A**) Fraction of inner-bound water (*f*_IBW_) as a function of COL concentration. The fractions were obtained from FTIR spectra. The line came from fitting with (6] resulting in α = 0.43, *n *=* *0.509, *R*^2^ = 0.99. (**B**) Comparison of the theoretically derived *T*_2_ values with the experimental *T*_2_ values with a squared correlation coefficient *R*^2^ = 0.86. (**C**) The attenuation signals at different concentrations of COL, which are contributed from IBW, OBW and FW (actually self-bound water).

Our experimental data fit this equation well, as seen in Fig. 6A. The fitted index *n* read 0.509, which is indeed <2/3, and has in turn confirmed the basic assumption of partial overlap of bound water when COL concentration is increased. For the convenience of subsequent derivation, we take *n *=* *0.5 in the later analysis.

The OBW content is closely related to the IBW. For simplicity, the proportional coefficient is set as β:


(7) $${f_{\mathrm{OBW}}\;={\beta f}_{\mathrm{IBW}}=\;\alpha \beta [\mathrm{COL}]}^{0.5}.$$

The percentage of FW is calculated as

(8) $$f_{\mathrm{FW}}=1-f_{\mathrm{IBW}}-f_{\mathrm{OBW}}.$$

In this article, the reduced MR signal of the COL-water system can be expressed as follows:


(9) $$M\left(t\right)={\;\alpha \left[\mathrm{COL}\right]}^{0.5}\exp\left(-\frac{t}{\tau_{\mathrm{IBW}}}\right)+{\;\alpha \beta \left[\mathrm{COL}\right]}^{0.5}\exp\left(-\frac{t}{\tau_{\mathrm{OBW}}}\right)+(1-{\;\alpha \left[\mathrm{COL}\right]}^{0.5}-{\;\alpha \beta [\mathrm{COL}]}^{0.5})\exp(-t/\tau_{\mathrm{FW}}).$$

It is known that


(10) $$M(T_{2})\equiv M\left(0\right)/e.\;$$

Then, we obtain


(11) $$\begin{array}{c} \left[\mathrm{COL}\right]=\{[1-\exp\left(1-\frac{T_{2}}{\tau_{\mathrm{FW}}}\right)]/\alpha [\exp\left(1-\frac{T_{2}}{\tau_{\mathrm{IBW}}}\right)-\exp\left(1-\frac{T_{2}}{\tau_{\mathrm{FW}}}\right)]\\ {+\;\alpha \beta [\exp\left(1-\frac{T_{2}}{\tau_{\mathrm{OBW}}}\right)-\exp\left(1-\frac{T_{2}}{\tau_{\mathrm{FW}}}\right)]\}}^{2} \end{array}.$$

Equation (11) quantifies well the results in Fig. 6B. The fitted β read 5.6, indicating that each IBW leads to an average of 5.6 OBW molecules. The final normalized MR signals based on the theory are shown in Fig. 6C.

### Effect of biomacromolecule content on *T*_1_ values

**Figure 7.**
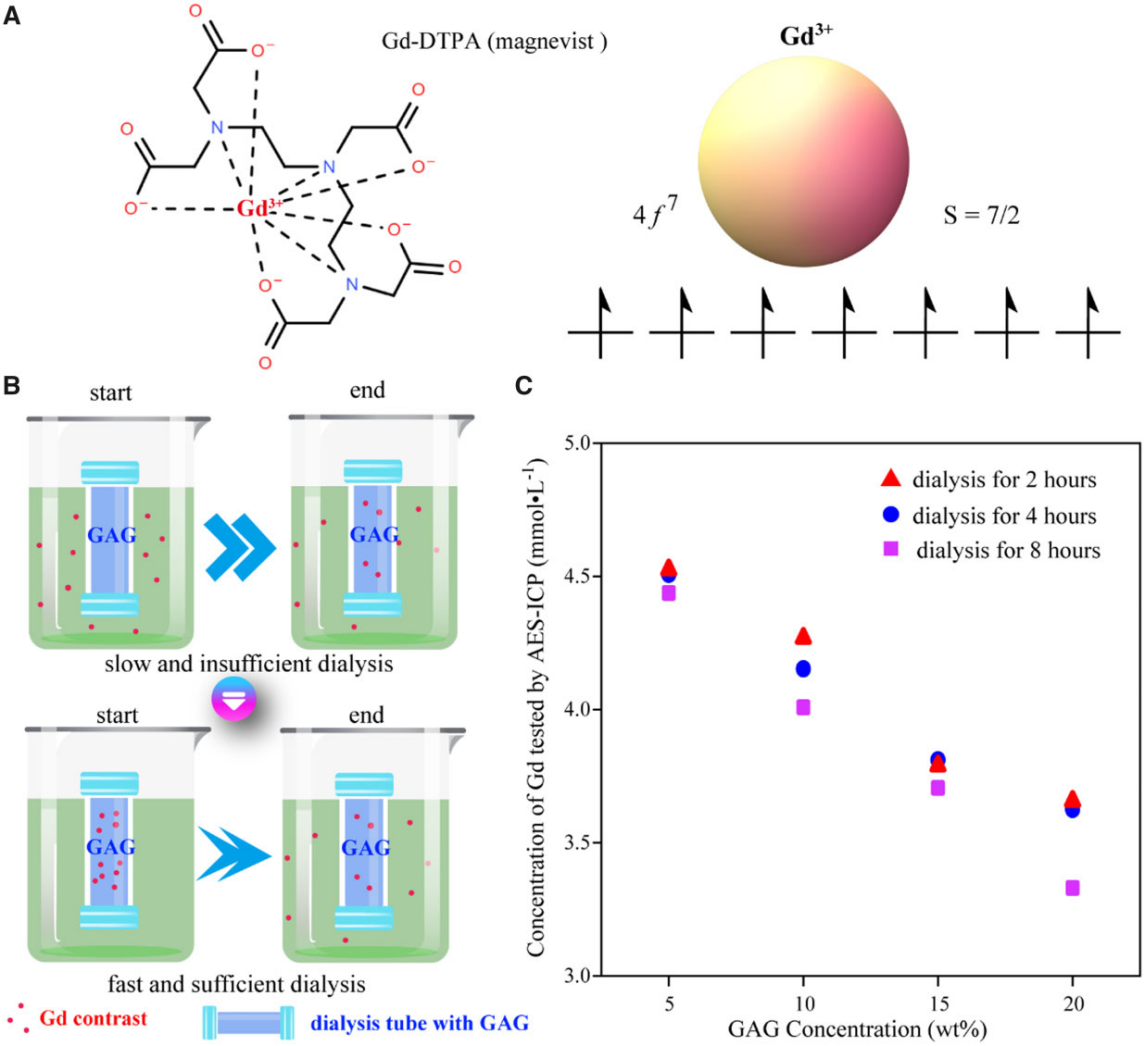
Content of a gadolinium contrast agent as a function of the content of GAG as demonstrated from a dialysis experiment. (**A**) Structural formulas of Gd-DTPA^2−^, the gadolinium contrast agent used in this study. (**B**) Schematic representation of dialysis processes of a gadolinium contrast agent to simulate its penetration into cartilage *in vivo*. The dialysis bags were filled with GAG in PBS (pH 7.4). The dialysis process is slow when the external gadolinium ions permeate into the dialysis bags and relatively fast when the gadolinium ions were initially added into the GAG solution, which we used to achieve rapid equilibrium. (**C**) Equilibrium gadolinium content as a function of GAG concentration. While gadolinium ions in the dialysis bag decreased significantly with the increase of GAG content, there was no significant difference in the concentration of remaining gadolinium after 2, 4 and 8 h of dialysis.

The clinical *in vivo* permeation of the gadolinium contrast agent was simulated with an *in vitro* dialysis method as shown in Fig. 7A. Specific dialysis tubes (Float-A-Lyzer G2, Spectrumlabs) were selected, which can open the knob at any time for sampling and testing. The pore size of the dialysis tube only allowed the penetration of gadolinium ions and did not allow the migration of GAG macromolecules.

The gadolinium contrast agent used in this study was Gd-DTPA^2−^ (Magenvist, Bayer). The Gd^3+^ ion has seven electrons in its 4*f* orbital and has the highest spin value in the periodic table with total spin *S* = 7/2. The interaction between free spin electrons in the Gd contrast agent and protons significantly affects proton relaxation, which influences both *T*_1_ and *T*_2_. Since *T*_1_ is usually much larger than *T*_2_, the effect of the contrast agent on *T*_1_ is more pronounced.

Our dialysis protocol I mimics cartilage with different GAG contents, and the gadolinium agent was added to the extra-dialysis fluid to simulate the intravenously injected contrast agent. The actual dialysis was slow and the driving force was found insufficient. So, we suggested dialysis protocol II, as shown in Fig. 7B. Here, both negatively charged gadolinium contrast agent and GAG were initially inside the dialysis tube, and the pore size of the dialysis tube allowed only the penetration of the gadolinium contrast agent without affecting the biomolecular concentration. The concentration of the remaining contrast agent inside the dialysis bag was finally examined. Dialysis protocol II was subsequently selected as the final protocol for dialysis simulation with results shown in Fig. 7C.

The addition of GAG exhibited significant effects on the dialysis of Gd-DTPA^2−^. The data also indicate that dialysis extravasation mainly occurred in the first 2 h, which is consistent with the time of injection of the contrast agent in clinical patients.

As for the detection of the gadolinium contrast agent, we tried two methods, UV–visible spectroscopy and ICP-AES, as shown in Supplementary Fig. S10. In the UV–visible spectrum scanning, both Gd-DOTA and Gd-DTPA gadolinium contrast agents exhibited obvious absorption peaks at a specific wavelength (274 nm), and the peak intensity is linearly related to the concentration. The disadvantage of the UV–vis scanning method is the detection concentration limit, which makes it difficult to accurately determine the gadolinium contrast agents with micromolar concentrations. So, we further employed ICP-AES to determine the concentration of Gd. Although the standard curve has to be remade for each ICP-AES test, which is thus a bit tedious, the linearity of the standard curve obtained in each of our tests is excellent (*R*^2^ close to 1), and low concentrations are detectable.

### Effect of concentrations of biomacromolecules on *T*_1_

**Figure 8.**
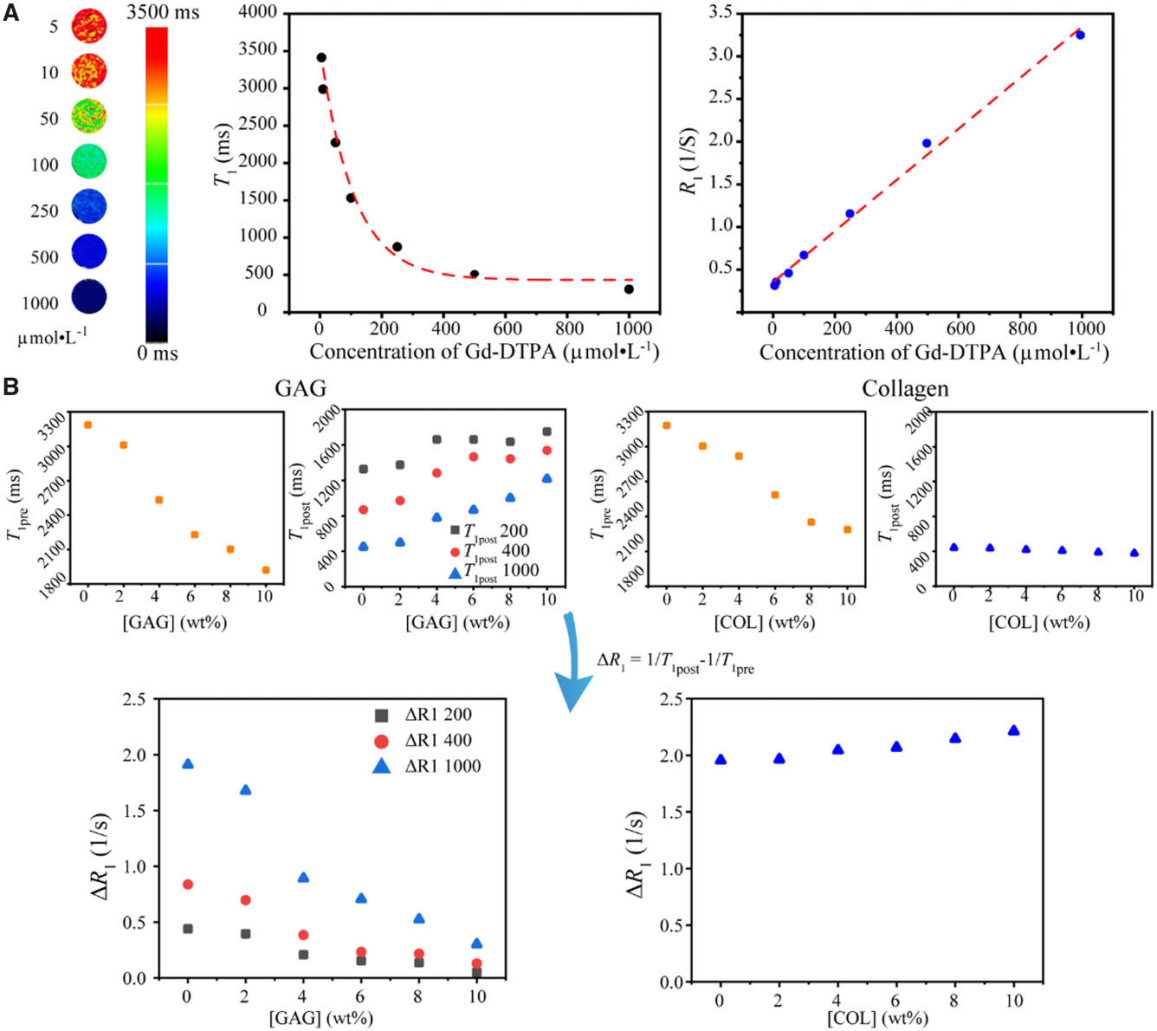
Establish the relation of GAG concentrations and *T*_1_-weighted mapping relevant quantities. (**A**) Lateral relaxation time *T*_1_ and the corresponding relaxation velocity *R*_1_ as a function of gadolinium contrast agent. Here, neither GAG nor COL was added. (**B**) The difference in GAG concentration can significantly affect the dialysis of gadolinium contrast medium and ultimately affect the measured *T*_1_ and Δ*R*_1_ values, while the COL concentration had little effect on the dialysis of the gadolinium contrast. The initial concentrations of gadolinium in dialysis bags containing GAG were 200, 400 and 1000 µmol/l. And the initial concentrations of gadolinium in dialysis bags containing COL were 1000 µmol/l.

The relaxation time *T*_1_ decreases significantly as the concentration of gadolinium contrast agent increases, as shown in Fig. 8A. In particular, we took the inverse of the relaxation time *T*_1_ as the relaxation rate *R*_1_ and found a significant positive correlation between *R*_1_ with the content of the gadolinium contrast agent.

Different concentrations of GAG solutions were prepared, and three batches of dialysis experiments were performed with the addition of the gadolinium contrast agent. According to the experience of the above-mentioned dialysis experiments, dialysis for 2 h can achieve a relatively sufficient penetration effect of the gadolinium contrast agent. The *T*_1_ values were measured before and after the addition of the gadolinium contrast agent, and the relaxation rate difference Δ*R*_1_ was calculated, as shown in Fig. 8B.

It can be found that the presence of GAG reduced Δ*R*_1_ significantly. Since both gadolinium contrast agent and GAG are negatively charged, the higher the GAG content, the less contrast agent remains in the dialysis tubing, resulting in a larger *T*_1_ post and smaller Δ*R*_1_.

To compare the possible effect of COL on *T*_1_ values, a series of COL solutions with different concentrations were additionally configured. We found that the effect of COL on *T*_1_ and Δ*R*_1_ after dialysis was very limited compared to that of GAG.

### A clinical case of MRI during cartilage regeneration

**Figure 9.**
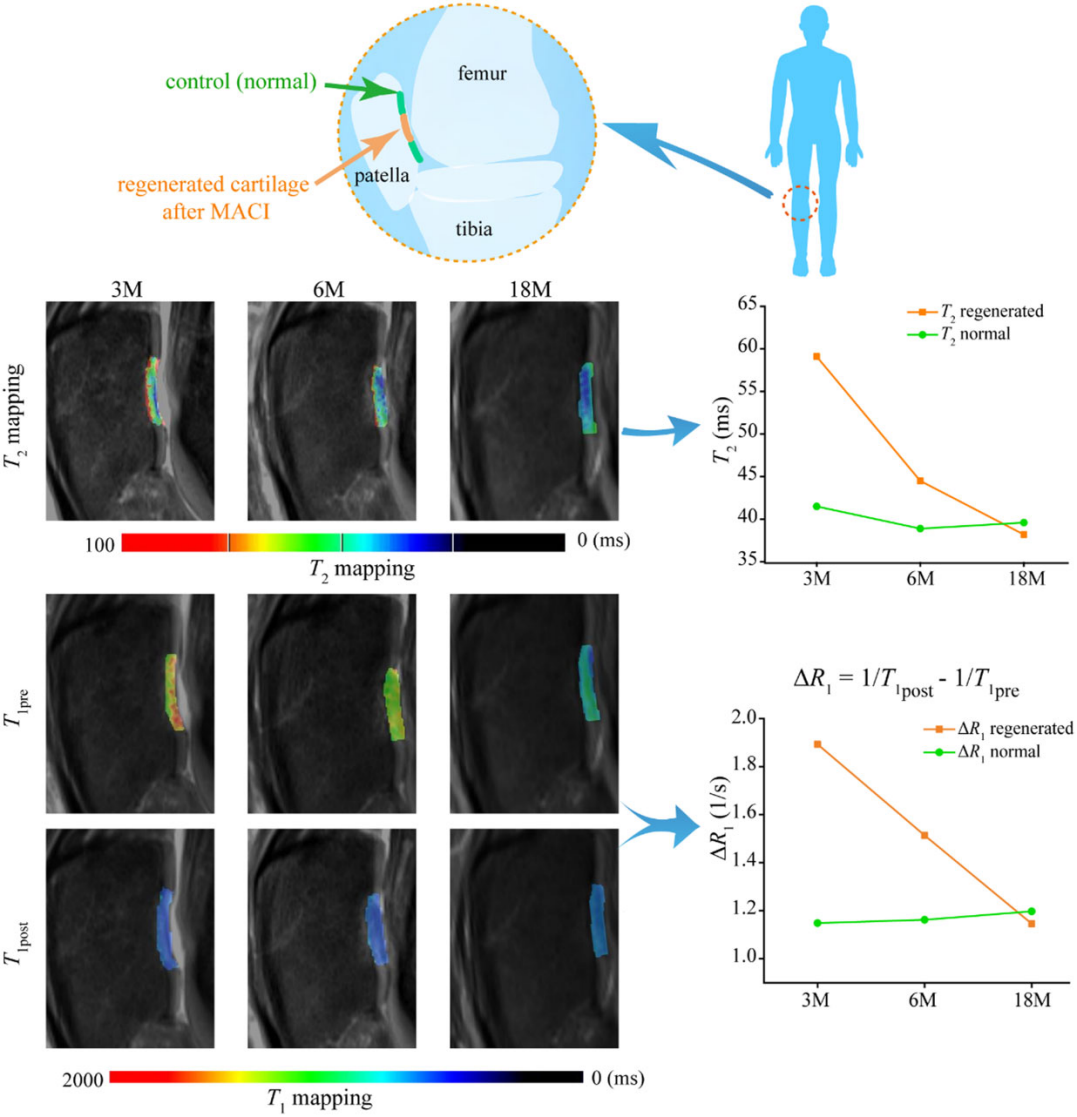
A clinical case of MRI for *T*_2_ and *T*_1_ mapping of the knee joint. The patient is male and 46 years old. His right patella cartilage was treated with implanting of MACI and detected after the indicated follow-up times. The line pictures show the clear decrease of *T*_2_ and Δ*R*_1_ in the regenerated tissue at 3 and 6 months, and similar values between the regenerated tissue and the control normal cartilage at 18 months.

A clinical case is reported to confirm the MRI method to monitor cartilage regeneration. Figure 9 shows typical MRI images of a male patient with patellar injury of the right knee at 3, 6 and 18 months after MACI surgery. This is from the cohort of our previous publication including 18 cases and 25 lesions [10], and the data in Fig. 9 for this case have not yet been published previously. To compare the difference between regenerated cartilage and healthy cartilage at different repair stages and the corresponding changes in MRI indexes, regions of morphologically normal cartilage in the adjacent anatomical region were selected as controls. The ROI of regenerated cartilage is manually drawn by experienced musculoskeletal radiologists. *T*_2_ and *T*_1_ values of both regenerated cartilage and adjacent normal tissue were measured.

Compared with the surrounding normal cartilage area, the *T*_2_ and Δ*R*_1_ values of regenerated cartilage tissue were higher than those of normal tissue at 3 and 6 months after the operation. After 18 months, there was no significant difference between the two groups, illustrating the regeneration efficacy. These clinical imaging data demonstrate the feasibility of non-invasive quantitative evaluation of cartilage regeneration by MRI.

## Discussion

While much progress has been made for biomedical materials [35–42], cell–material interactions [43–46] and medical devices [47–52] in the latest decade, it is rare to find a non-invasive approach to monitor *in vivo* cartilage regeneration, which is particularly meaningful for clinical research after implanting a regenerative biomaterial. The histological evaluation of arthroscopic biopsies provides a gold standard for morphological and biochemical evaluation of regenerated cartilage tissue. However, this process is invasive and difficult to be accepted by patients. In recent years, MRI has increasingly been an important means of disease assessment [53–57]. The quantification of MRI indexes can reduce the subjectivity encountered by traditional non-quantitative techniques. The relaxation signal of tissue is measured in MRI, of which the main factors affecting the relaxation rate include water content, system viscosity and the effect of paramagnetic substances [16, 58–60]. In this study, *T*_2_ and *T*_1_ mapping were used as quantitative methods to determine the regeneration effect of cartilage tissue.

Some researchers have reported the influence of MRI indexes on the intra-cartilage components, including water [16, 61], COL [17, 62, 63] and GAG [15, 64] during the occurrence of osteoarthritis or the regeneration of cartilage. However, the reason underlying the effect is still vague. Even some reports are contradictory with each other; for instance, it is not clear whether the *T*_2_ value of cartilage tissue is independent of proteoglycans [13, 14] or affected by proteoglycans in cartilage [65, 66]. Therefore, it is necessary to make a deterministic experiment to examine the effects of specific cartilage components on *T*_1_ and *T*_2_ in a ‘clean’ system without the interference of other biomacromolecules and to reveal the underlying physical principle.

**Figure 10.**
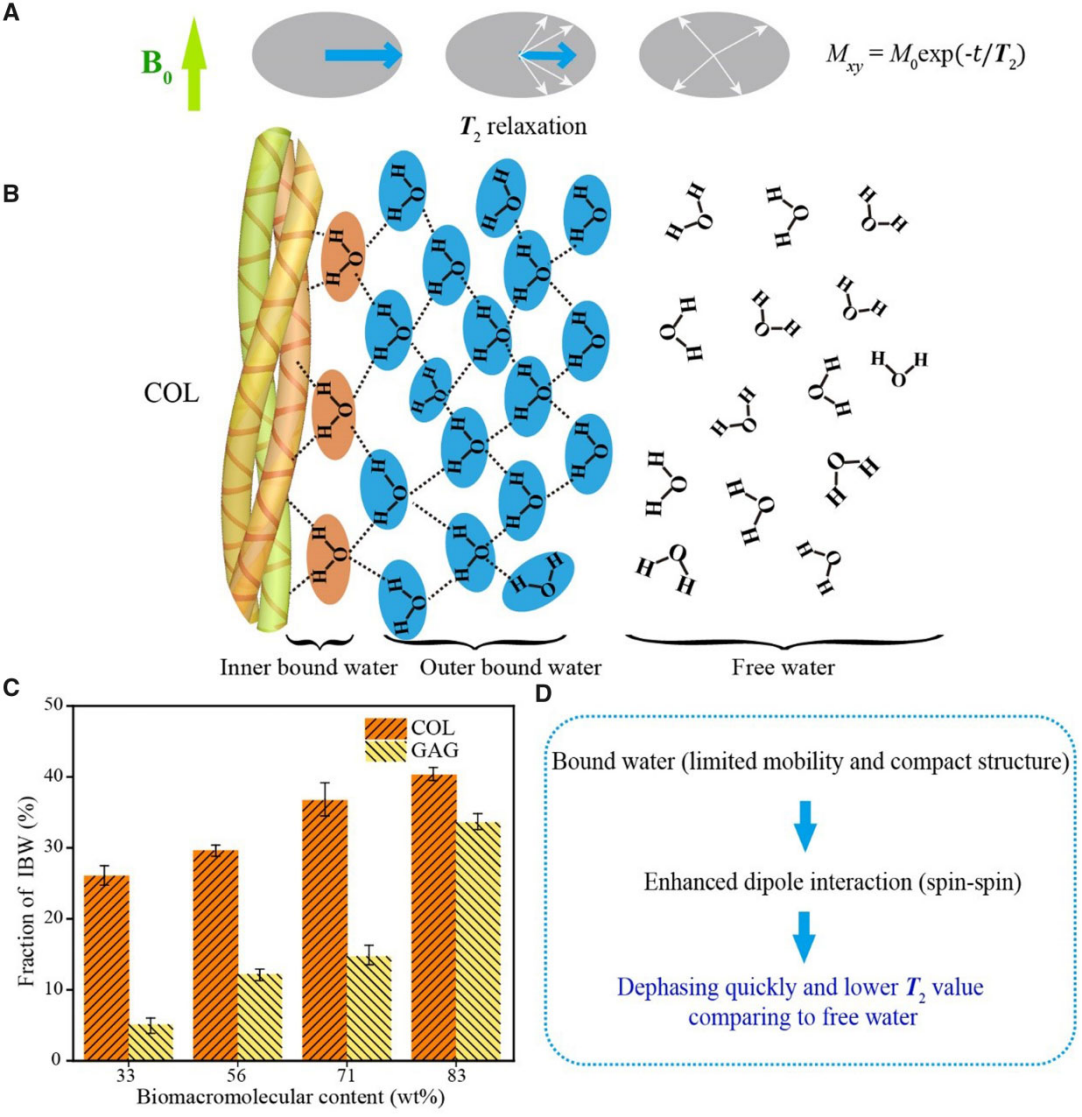
Analysis of main factors affecting *T*_2_ values of cartilage. (**A**) Schematic presentation to indicate the nature of *T*_2_ in the dephasing process caused by spin–spin interaction. (**B**) Schematic presentation to indicate the influence of COL as a biomacromolecule on the water form. Here, we divide the bound water into inner-bound water and outer-bound water. The so-called FW is actually a network of water with hydrogen bonds as well, and the schematic way is simply to emphasize their mobility. The inner-bound water content can be determined from the fitting of the infrared spectra, and the content of outer-bound water can be quantified from MR fitting with an (11] based on the data of *T*_2_ versus [COL]. (**C**) The experimental fractions of inner-bound water (*n *=* *3). The biological macromolecules significantly affect the bound water and thus the *T*_2_ values. Although both COL and GAG in cartilage affect the fraction of bound water, COL has a greater impact and plays a major role in regulating *T*_2_ values. (**D**) Summary of the mechanism of [COL] effects on MR lateral relaxation. The biomacromolecules influence bound water and ultimately the *T*_2_ values.

NMR can respond to specific atomic nuclei, reflecting the structure and composition of matter [67–69]. The MRI signal in this study reflects the relaxation of protons, which is mainly contributed by the hydrogen nuclei in the water molecules. We suggest that the measured *T*_2_ value is related to the relative contents of bound water and FW, which are greatly influenced by biomacromolecules in cartilage. In this study, we combine theoretical derivation and *in vitro* experimental tests to conclude unambiguously that it is COL that mainly influences *T*_2_ values. COL biomacromolecules affect the activity and spin–spin dipole interaction of its bound water, and this spin–spin dipole interaction between protons is reflected in the *T*_2_ mapping, as summarized in Fig. 10.

Combining the IR tests and the data on the bound water content by Gaussian peak splitting, the effect of COL is more significant than that of GAG on the bound water content, and thus the *T*_2_ value of the MRI reflects more COL content of the articular cartilage tissue. Based on the IR measurement and analysis of curve fitting of bound water content, we have revealed that the effect of COL on bound water content is greater than that of GAG. In theory, the orientation of COL also has a certain influence on the *T*_2_ signal [63, 70], which has not been considered in this study. A highlight of the present study is the concept and mathematical treatment of OBW, as schematically presented in Fig. 10B.

MRI measures the relaxation of hydrogen protons of water molecules in tissue, where the relaxation of water molecules is slow, resulting in large *T*_1_ and *T*_2_ values. The large *T*_2_ is easy to detect and leads to a high signal along with slow transverse relaxation. However, a large *T*_1_ value indicates a slow recovery of longitudinal relaxation, and therefore the signal is very weak. The commonly used method for detecting *T*_1_ in clinic is dGEMRIC, where a gadolinium contrast agent is added to accelerate *T*_1_ relaxation and thus enhance the *T*_1_ signal. Bashir *et al.* [12] first reported the effect of an ionic gadolinium contrast agent on the enhancement of cartilage MRI. Later, a few reports suggested that the evaluation of contrast agents on regenerated cartilage was unnecessary [71]. Yet, most of the pertinent publications about dGEMRIC supported that the relaxation rate difference Δ*R*_1_ before and after intravenous injection of contrast agent was associated with GAG content [12, 72, 73]. Watanabe *et al.* [74] reported that *R*_1pre_ (1/*T*_1pre_) was not significantly correlated with relative GAG concentration and Δ*R*_1_ was significantly negatively correlated with GAG concentration. Our experimental results (Fig. 8) have illustrated that all of *T*_1pre_, *T*_1post_ and Δ*R*_1_ are sensitive to [GAG], and it is Δ*R*_1_ that distinguishes GAG from COL the best.

The premise that the gadolinium contrast agent can effectively evaluate cartilage tissue is a clear inverse correlation between gadolinium contrast agent and GAG concentration. To verify that we *in vitro* used a dialysis device to simulate the permeation process of contrast medium and investigated the effects of different concentrations of GAG on contrast medium penetration and further MRI quantitative parameters *T*_1_. Due to the negative charge of GAG and gadolinium contrast medium, the distribution of Gd-DTPA^2−^ in cartilage is negatively correlated with the content of GAG, which can indirectly reflect the maturity of cartilage [73, 75–77]. Gd-DTPA^2−^ is concentrated in the areas with low GAG content, speeding up *T*_1_ relaxation in these areas. Kurkijarvi *et al.* [78] reported that there was no significant difference in dGEMRIC assessment between the regenerated tissue and the control cartilage 5–10 months after surgery, but they did not perform short-term follow-up. Gillis *et al.* [79] believed that the concentration of GAG in the regenerated cartilage 12 months or longer after ACI was similar to that in the surrounding normal cartilage, which our clinical MRI has supported well.

**Figure 11.**
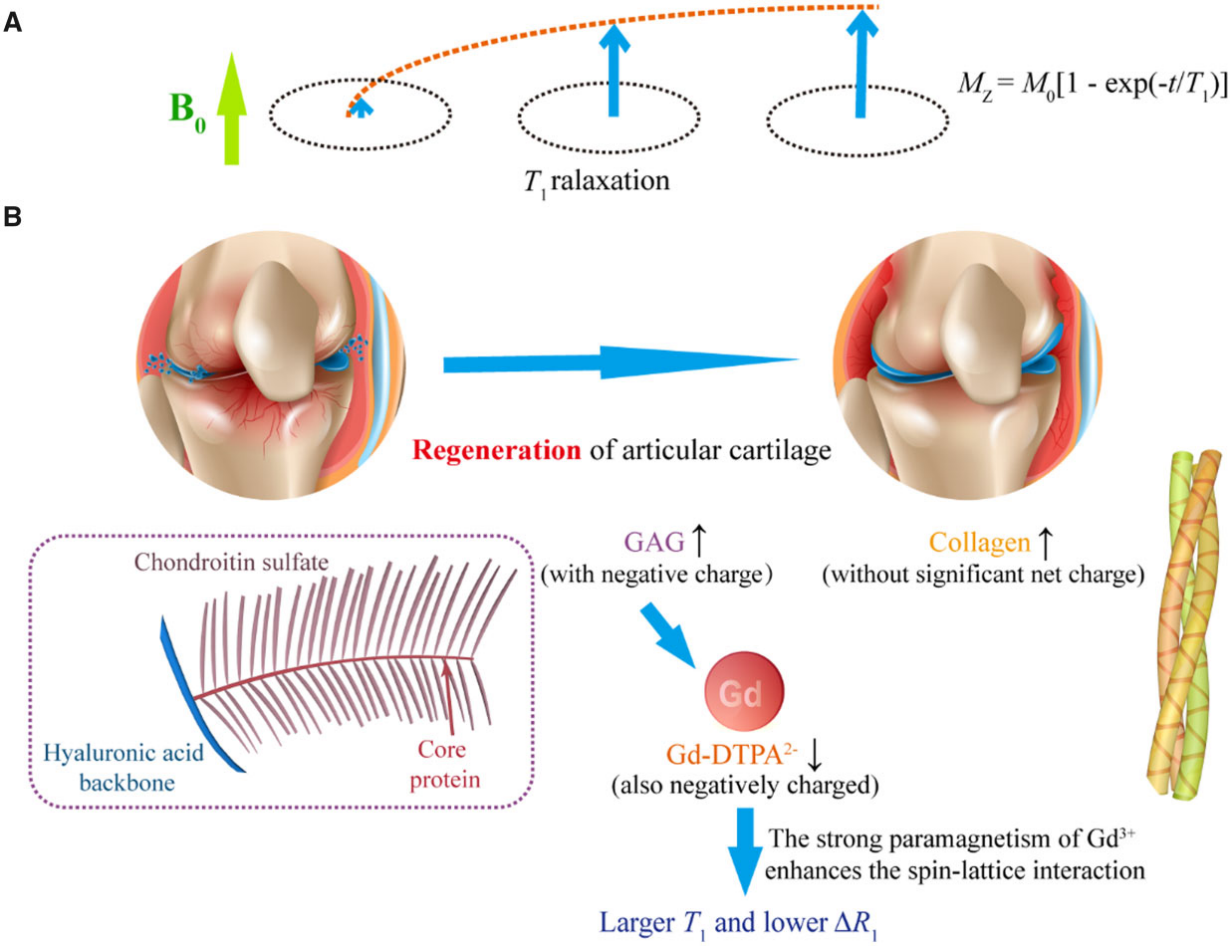
Analysis of main factors affecting *T*_1_ values of cartilage. (**A**) The nature of *T*_1_ lies in the recovery of the longitudinal magnetic moment caused by spin–lattice interaction. (**B**) Schematic diagram of GAG effect on *T*_1_ mapping. The gadolinium contrast agent can indicate the GAG content in cartilage by reflecting the negative charge density in cartilage tissue indirectly.

In our clinical evaluation, 90–120 min after contrast agent administration was selected as the time window for dGEMRIC imaging, which was consistent with previous reports [75, 80]. We found that Δ*R*_1_ in the regenerated tissue was significantly higher than that in the control normal cartilage at 3 and 6 months, which is reasonable that the GAG content in the regenerated cartilage was lower than that in the normal cartilage. As shown in Fig. 11, this study has confirmed the rationality of using the Gd contrast agent to characterize GAG *in vitro* and in cartilage tissue *in vivo* and verified that *T*_1_ mapping before and after the addition of gadolinium contrast agent can quantitatively reflect the spatiotemporal distribution of GAG.

## Conclusions

This work has established the definite relations of MR indexes *T*_1_ and *T*_2_ to the contents of the two main biomacromolecules in cartilage ECM based on ‘clean’ systems *in vitro*. The MRI signal in the biomacromolecule-water system is thought to be affected by the hydrogen proton of the biomacromolecule-bound water. In *T*_2_-weighted imaging, COL exhibited more significant effects on *T*_2_ relaxation than GAG, which has been interpreted from the fact that COL generates more the IBW and subsequently the OBW. GAG can regulate the distribution of the negatively charged gadolinium contrast agent in cartilage tissue owing to the charge effect and thus significantly affect *T*_1_-weighted imaging. In addition, this work reports the quantitative clinical analysis of MRI on the regeneration of human knee cartilage 18 months after MACI surgery. Through *T*_2_ mapping and dGEMRIC methods, the values of *T*_2_ and Δ*R*_1_ were found to decrease gradually at 3, 6 and 12 months after MACI, indicating the maturation of the COL network and the increase of the GAG content in the regenerated cartilage. This study affords one of the academic bases of MRI standards to evaluate cartilage regeneration *in vivo* in a real-time and non-invasive manner. The fundamental research has ever greatly supported the suggestion of an ISO standard by the Chinese team, which was approved as ISO/TS24560-1:2002 in September 2022.

In addition, the present study has revealed two basic relations: one is between biomacromolecule content and IBW and the other is between the content of bound water and the characteristic MR relaxation time. The content of IBW can be estimated from Fourier transform infrared spectrometry, and the ratio between OBW and IBW can be estimated further from MRI data. We have discovered that one IBW can lead to 5.6 OBWs in the examined system. The concept and the methodology are helpful for the quantitative investigation of the aqueous systems containing biomacromolecules in tissue ECM including but not necessarily limited to COL and GAG in cartilage.

## Supplementary Material

rbad035_Supplementary_Data

## References

[1] Huey DJ , Hu JC, Athanasiou KA. Unlike bone, cartilage regeneration remains elusive. Science 2012;338:917–21. 23161992 10.1126/science.1222454 PMC4327988

[2] Mastbergen SC , Saris DB, Lafeber FP. Functional articular cartilage repair: here, near, or is the best approach not yet clear?. Nat Rev Rheumatol 2013;9:277–90. 23507899 10.1038/nrrheum.2013.29

[3] Makris EA , Gomoll AH, Malizos KN, Hu JC, Athanasiou KA. Repair and tissue engineering techniques for articular cartilage. Nat Rev Rheumatol 2015;11:21–34. 25247412 10.1038/nrrheum.2014.157 PMC4629810

[4] Zhou LB , Van Osch GJVM, Malda J, Stoddart MJ, Lai YX, Richards RG, Ho KKW, Qin L. Innovative tissue-engineered strategies for osteochondral defect repair and regeneration: current progress and challenges. Adv Healthcare Mater 2020;9:2001008. 10.1002/adhm.202001008 33103381

[5] Cao DLG , Ding JD. Recent advances in regenerative biomaterials. Regen Biomater 2022;9:rbac098. 36518879 10.1093/rb/rbac098 PMC9745784

[6] Mankin HJ , Dorfman H, Lippiello L, Zarins A. Biochemical and metabolic abnormalities in articular cartilage from osteo-arthritic human hips. II. Correlation of morphology with biochemical and metabolic data. J Bone Joint Surg 1971;53:523–37. 5580011

[7] Outerbridge RE. The etiology of chondromalacia patellae. J Bone Joint Surg Br 1961;43-B:752–7. 14038135 10.1302/0301-620X.43B4.752

[8] Benyley G , Dowd G. Current concepts of etiology and treatment of chondromalacia patellae. Clin Orthop Relat Res 1984;189:209–28. 6383677

[9] Ficat RP , Philippe J, Hungerford DS. Chondromalacia patellae—system of classification. Clin Orthop Relat Res 1979;144:55–62. 535251

[10] Xu X , Gao JM, Liu SY, Chen L, Chen M, Yu XY, Ma N, Zhang J, Chen XB, Zhong LS, Yu L, Xu LM, Guo QY, Ding JD. Magnetic resonance imaging for non-invasive clinical evaluation of normal and regenerated cartilage. Regen Biomater 2021;8:rbab038. 34408910 10.1093/rb/rbab038 PMC8369076

[11] Bloch F , Hansen WW, Packard M. The nuclear induction experiment. Phys Rev 1946;70:474–85.

[12] Bashir A , Gray ML, Boutin RD, Burstein D. Glycosaminoglycan in articular cartilage: in vivo assessment with delayed Gd(DTPA)^2-^-enhanced MR imaging. Radiology 1997;205:551–8. 9356644 10.1148/radiology.205.2.9356644

[13] Fragonas E , Mlynarik V, Jellus V, Micali F, Piras A, Toffanin R, Rizzo R, Vittur F. Correlation between biochemical composition and magnetic resonance appearance of articular cartilage. Osteoarthr Cartil 1998;6:24–32. 10.1053/joca.1997.0089 9616436

[14] Nieminen MT , Töyräs J, Rieppo J, Hakumäki JM, Silvennoinen J, Helminen HJ, Jurvelin JS. Quantitative MR microscopy of enzymatically degraded articular cartilage. Magn Reson Med 2000;43:676–81. 10800032 10.1002/(sici)1522-2594(200005)43:5<676::aid-mrm9>3.0.co;2-x

[15] Watrin-Pinzano A , Ruaud JP, Olivier P, Grossin L, Gonord P, Blum A, Netter P, Guillot G, Gillet P, Loeuille D. Effect of proteoglycan depletion on T2 mapping in rat patellar cartilage. Radiology 2005;234:162–70. 15564387 10.1148/radiol.2341030394

[16] Lusse S , Claassen H, Gehrke T, Hassenpflug J, Schunke M, Heller M, Gluer CC. Evaluation of water content by spatially resolved transverse relaxation times of human articular cartilage. Magn Reson Imaging 2000;18:423–30. 10788720 10.1016/s0730-725x(99)00144-7

[17] Xia Y. Relaxation anisotropy in cartilage by NMR microscopy (μMRI) at 14-μm resolution. Magn Reson Med 1998;39:941–9. 9621918 10.1002/mrm.1910390612

[18] Jiang SP , Guo WM, Tian GZ, Luo XJ, Peng LQ, Liu SY, Sui X, Guo QY, Li X. Clinical application status of articular cartilage regeneration techniques: tissue-engineered cartilage brings new hope. Stem Cells Int 2020;2020:5690252. 32676118 10.1155/2020/5690252 PMC7345961

[19] Behrens P , Bitter T, Kurz B, Russlies M. Matrix-associated autologous chondrocyte transplantation/implantation (MACT/MACI)—5-year follow-up. Knee 2006;13:194–202. 16632362 10.1016/j.knee.2006.02.012

[20] Zheng MH , Willers C, Kirilak L, Yates P, Xu JK, Wood D, Shimmin A. Matrix-induced autologous chondrocyte implantation (MACI): biological and histological assessment. Tissue Eng 2007;13:737–46. 17371156 10.1089/ten.2006.0246

[21] Perakis F , De Marco L, Shalit A, Tang FJ, Kann ZR, Kuhne TD, Torre R, Bonn M, Nagata Y. Vibrational spectroscopy and dynamics of water. Chem Rev 2016;116:7590–607. 27096701 10.1021/acs.chemrev.5b00640

[22] Li L , Yu YF, Su HH, Zhan GZ, Li SJ, Wu PY. The diffusion mechanism of water transport in amine-cured epoxy networks. Appl Spectrosc 2010;64:458–65. 20412632 10.1366/000370210791114220

[23] Kawamoto T , Ochiai S, Kagi H. Changes in the structure of water deduced from the pressure dependence of the Raman OH frequency. J Chem Phys 2004;120:5867–70. 15267467 10.1063/1.1689639

[24] Freda M , Piluso A, Santucci A, Sassi P. Transmittance Fourier transform infrared spectra of liquid water in the whole mid-infrared region: temperature dependence and structural analysis. Appl Spectrosc 2005;59:1155–9. 18028611 10.1366/0003702055012591

[25] Masuda K , Haramaki T, Nakashima S, Habert B, Martinez I, Kashiwabara S. Structural change of water with solutes and temperature up to 100 degrees in aqueous solutions as revealed by attenuated total reflectance infrared spectroscopy. Appl Spectrosc 2003;57:274–81. 14658618 10.1366/000370203321558173

[26] Raichlin Y , Millo A, Katzir A. Investigations of the structure of water using mid-IR fiberoptic evanescent wave spectroscopy. Phys Rev Lett 2004;93:185703. 15525181 10.1103/PhysRevLett.93.185703

[27] Schmidt DA , Miki K. Structural correlations in liquid water: a new interpretation of IR spectroscopy. J Phys Chem A 2007;111:10119–22. 17880191 10.1021/jp074737n

[28] Nakamoto K , Margoshes M, Rundle RE. Stretching frequencies as a function of distances in hydrogen bonds. J Am Chem Soc 1955;77:6480–6.

[29] Kudo S , Nakashima S. Water retention capabilities of collagen, gelatin and peptide as studied by IR/QCM/RH system. Spectrochim Acta A: Mol Biomol Spectrosc 2020;241:118619. 32622049 10.1016/j.saa.2020.118619

[30] Kudo S , Ogawa H, Yamakita E, Watanabe S, Suzuki T, Nakashima S. Adsorption of water to collagen as studied using infrared (IR) microspectroscopy combined with relative humidity control system and quartz crystal microbalance. Appl Spectrosc 2017;71:1621–32. 28664780 10.1177/0003702817693855

[31] Susi H. Infrared spectroscopy—conformation. In: Hirs CHW, Timasheff SN (eds). Methods in Enzymology, Vol. 26. Cambridge, Massachusetts: Academic Press, 1972, 455–472. 4680721 10.1016/s0076-6879(72)26024-4

[32] Leikin S , Parsegian VA, Yang W-H, Walrafen GE. Raman spectral evidence for hydration forces between collagen triple helices. Proc Natl Acad Sci U S A 1997;94:11312–7. 9326606 10.1073/pnas.94.21.11312 PMC23454

[33] Laule C , Vavasour IM, Kolind SH, Li DKB, Traboulsee TL, Moore GRW, MacKay AL. Magnetic resonance imaging of myelin. Neurotherapeutics 2007;4:460–84. 17599712 10.1016/j.nurt.2007.05.004 PMC7479725

[34] Zhang J , Kolind SH, Laule C, MacKay AL. Comparison of myelin water fraction from multiecho T2 decay curve and steady-state methods. Magn Reson Med 2015;73:223–32. 24515972 10.1002/mrm.25125

[35] Peters JT , Wechsler ME, Peppas NA. Advanced biomedical hydrogels: molecular architecture and its impact on medical applications. Regen Biomater 2021;8:rbab060. 34925879 10.1093/rb/rbab060 PMC8678442

[36] Gao JM , Yu X, Wang X, He Y, Ding J. Biomaterial-related cell microenvironment in tissue engineering and regenerative medicine. Engineering 2022;13:31–45.

[37] Ding X , Gao JM, Yu X, Shi J, Chen J, Yu L, Chen S, Ding J. 3D-printed porous scaffolds of hydrogels modified with TGF-β1 binding peptides to promote *in vivo* cartilage regeneration and animal gait restoration. ACS Appl Mater Interfaces 2022;14:15982–95. 35363484 10.1021/acsami.2c00761

[38] Gao JM , Ding XQ, Yu XY, Chen XB, Zhang XY, Cui SQ, Shi JY, Chen J, Yu L, Chen SY, Ding JD. Cell-free bilayered porous scaffolds for osteochondral regeneration fabricated by continuous 3D-printing using nascent physical hydrogel as ink. Adv Healthcare Mater 2021;10:2001404. 10.1002/adhm.202001404 33225617

[39] Yu Y , Wang XL, Zhu Y, He YN, Xue HR, Ding JD. Is polydopamine beneficial for cells on the modified surface?. Regen Biomater 2022;9:rbac078. 36324608 10.1093/rb/rbac078 PMC9610633

[40] Pan Z , Duan PG, Liu XN, Wang HR, Cao L, He Y, Dong J, Ding JD. Effect of porosities of bilayered porous scaffolds on spontaneous osteochondral repair in cartilage tissue engineering. Regen Biomater 2015;2:9–19. 26813511 10.1093/rb/rbv001 PMC4669027

[41] Cao DLG , Guo W, Cai CY, Tang JY, Rao WH, Wang Y, Wang YB, Yu L, Ding JD. Unified therapeutic-prophylactic vaccine demonstrated with a postoperative filler gel to prevent tumor recurrence and metastasis. Adv Funct Mater 2022;32:2206084.

[42] Dong LP , Liu QL, Gao YL, Jia HX, Dai WL, Guo LK, Fan HS, Fan YJ, Zhang XD. The effect of collagen hydrogels on chondrocyte behaviors through restricting the contraction of cell/hydrogel constructs. Regen Biomater 2021;8:rbab030. 34221449 10.1093/rb/rbab030 PMC8245754

[43] Shen Y , Zhang WQ, Xie YM, Li AN, Wang XL, Chen XM, Liu QS, Wang QS, Zhang G, Liu Q, Liu JX, Zhang DW, Zhang ZY, Ding JD. Surface modification to enhance cell migration on biomaterials and its combination with 3D structural design of occluders to improve interventional treatment of heart diseases. Biomaterials 2021;279:121208. 34749074 10.1016/j.biomaterials.2021.121208

[44] Peng YM , Liu QJ, He TL, Ye K, Yao X, Ding JD. Degradation rate affords a dynamic cue to regulate stem cells beyond varied matrix stiffness. Biomaterials 2018;178:467–80. 29685517 10.1016/j.biomaterials.2018.04.021

[45] Tang J , Peng R, Ding JD. The regulation of stem cell differentiation by cell-cell contact on micropatterned material surfaces. Biomaterials 2010;31:2470–6. 20022630 10.1016/j.biomaterials.2009.12.006

[46] Ye K , Wang X, Cao LP, Li SY, Li ZH, Yu L, Ding JD. Matrix stiffness and nanoscale spatial organization of cell-adhesive ligands direct stem cell fate. Nano Lett 2015;15:4720–9. 26027605 10.1021/acs.nanolett.5b01619

[47] Zhang HJ , Zhang WQ, Qiu H, Zhang G, Li X, Qi HP, Guo JZ, Qian J, Shi XL, Gao X, Shi DK, Zhang DY, Gao RL, Ding JD. A biodegradable metal-polymer composite stent safe and effective on physiological and serum-containing biomimetic conditions. Adv Healthcare Mater 2022;11:2201740. 10.1002/adhm.202201740 36057108

[48] Wang G , Gao C, Xiao B, Zhang J, Jiang X, Wang Q, Guo J, Zhang D, Liu J, Xie Y, Shu C, Ding JD. Research and clinical translation of trilayer stent-graft of expanded polytetrafluoroethylene for interventional treatment of aortic dissection. Regen Biomater 2022;9:rbac049. 35958517 10.1093/rb/rbac049 PMC9362767

[49] Yu X , Li G, Zheng Y, Gao J, Fu Y, Wang Q, Huang L, Pan X, Ding J. “Invisible” orthodontics by polymeric “clear” aligners molded on 3D-printed personalized dental models. Regen Biomater 2022;9:rbac007. 35414958 10.1093/rb/rbac007 PMC8992363

[50] Zhang JZ , Shang ZZ, Jiang YB, Zhang K, Li XG, Ma ML, Li YJ, Ma B. Biodegradable metals for bone fracture repair in animal models: a systematic review. Regen Biomater 2021;8:rbaa047. 33732493 10.1093/rb/rbaa047 PMC7947587

[51] Lopez-Marcial GR , Elango K, O'Connell GD. Addition of collagen type i in agarose created a dose-dependent effect on matrix production in engineered cartilage. Regen Biomater 2022;9:rbac048. 35991580 10.1093/rb/rbac048 PMC9390219

[52] Cai CY , Tang JY, Zhang Y, Rao WH, Cao DLG, Guo W, Yu L, Ding JD. Intelligent paper-free sprayable skin mask based on an in situ formed Janus hydrogel of an environmentally friendly polymer. Adv Healthcare Mater 2022;11:2102654. 10.1002/adhm.202102654 35286021

[53] Chen ZY , Sun B, Duan Q, Xue YJ, Zheng ES, He YY, Li GJ. Three-dimensional breath-hold MRCP using space pulse sequence at 3 T: comparison with conventional navigator-triggered technique. AJR Am J Roentgenol 2019;213:1247–52. 31386572 10.2214/AJR.19.21399

[54] Yang L , Fu S, Cai Z, Liu L, Xia C, Gong Q, Song B, Ai H. Integration of PEG-conjugated gadolinium complex and superparamagnetic iron oxide nanoparticles as T1–T2 dual-mode magnetic resonance imaging probes. Regen Biomater 2021;8:rbab064. 34881046 10.1093/rb/rbab064 PMC8648151

[55] Yan W , Xu X, Xu Q, Sun Z, Jiang Q, Shi D. Platelet-rich plasma combined with injectable hyaluronic acid hydrogel for porcine cartilage regeneration: a 6-month follow-up. Regen Biomater 2020;7:77–90. 32153994 10.1093/rb/rbz039 PMC7053269

[56] Cai JL , Chen GC, Jin RR, Deng CH, Huang SH, Yuan XX, Chen GJ, Zhao J, Wang ZY, Ai H. A core-shell polymeric-inorganic hybrid nanocomposite system for MRI-visible gene delivery application in cancer immunotherapy. J Ind Eng Chem 2019;76:188–96.

[57] Yu J , Yang C, Li JDS, Ding YC, Zhang L, Yousaf MZ, Lin J, Pang R, Wei LB, Xu LL, Sheng FG, Li CH, Li GJ, Zhao LY, Hou YL. Multifunctional Fe5C2 nanoparticles: a targeted theranostic platform for magnetic resonance imaging and photoacoustic tomography-guided photothermal therapy. Adv Mater 2014;26:4114–20. 24677251 10.1002/adma.201305811

[58] Ye Y , Liu X, Zhang Z, Wu Q, Jiang B, Jiang L, Zhang X, Liu M, Pielak GJ, Li C. (19) F NMR spectroscopy as a probe of cytoplasmic viscosity and weak protein interactions in living cells. Chemistry 2013;19:12705–10. 23922149 10.1002/chem.201301657

[59] Caravan P , Ellison JJ, McMurry TJ, Lauffer RB. Gadolinium(III) chelates as MRI contrast agents: structure, dynamics, and applications. Chem Rev 1999;99:2293–352. 11749483 10.1021/cr980440x

[60] Lutz AM , Seemayer C, Corot C, Gay RE, Goepfert K, Michel BA, Marincek B, Gay S, Weishaupt D. Detection of synovial macrophages in an experimental rabbit model of antigen-induced arthritis: ultrasmall superparamagnetic iron oxide-enhanced MR imaging. Radiology 2004;233:149–57. 15333767 10.1148/radiol.2331031402

[61] Liess C , Lusse S, Karger N, Heller M, Gluer CC. Detection of changes in cartilage water content using MRI T2-mapping *in vivo*. Osteoarthr Cartil 2002;10:907–13. 10.1053/joca.2002.0847 12464550

[62] Dunn TC , Lu Y, Jin H, Ries MD, Majumdar S. T2 relaxation time of cartilage at MR imaging: comparison with severity of knee osteoarthritis. Radiology 2004;232:592–8. 15215540 10.1148/radiol.2322030976 PMC4447089

[63] Nieminen MT , Rieppo J, Toyras J, Hakumaki JM, Silvennoinen J, Hyttinen MM, Helminen HJ, Jurvelin JS. T2 relaxation reveals spatial collagen architecture in articular cartilage: a comparative quantitative MRI and polarized light microscopic study. Magn Reson Med 2001;46:487–93. 11550240 10.1002/mrm.1218

[64] Keenan KE , Besier TF, Pauly JM, Han E, Rosenberg J, Smith RL, Delp SL, Beaupre GS, Gold GE. Prediction of glycosaminoglycan content in human cartilage by age, T1 rho and T2 MRI. Osteoarthr Cartil 2011;19:171–9. 10.1016/j.joca.2010.11.009 PMC3041640 21112409

[65] Nishioka H , Hirose J, Nakamura E, Oniki Y, Takada K, Yamashita Y, Mizuta H. T1 rho and T2 mapping reveal the in vivo extracellular matrix of articular cartilage. J Magn Reson Imaging 2012;35:147–55. 21990043 10.1002/jmri.22811

[66] Wong CS , Yan CH, Gong NJ, Li T, Chan QN, Chu YC. Imaging biomarker with T1 rho and T2 mappings in osteoarthritis in vivo human articular cartilage study. Eur J Radiol 2013;82:647–50. 23333531 10.1016/j.ejrad.2012.11.036

[67] Yang Y , Schuster M, Blumich B, Spiess HW. Dynamic magic-angle spinning NMR-spectroscopy–exchange-induced side-band. Chem Phys Lett 1987;139:239–43.

[68] Pan D , Wang LQ, Chen CH, Teng BS, Wang CD, Xu ZX, Hu BW, Zhou P. Structure characterization of a novel neutral polysaccharide isolated from ganoderma lucidum fruiting bodies. Food Chem 2012;135:1097–103. 22953830 10.1016/j.foodchem.2012.05.071

[69] Chen X , Huang DF, He LL, Li Z, Ren YH, Chen XY, Bin Y, He HY. Effect of adsorbed water molecules on the surface acidity of niobium and tantalum oxides studied by mas NMR. J Phys Chem C 2021;125:9330–41.

[70] Fullerton GD , Rahal A. Collagen structure: the molecular source of the tendon magic angle effect. J Magn Reson Imaging 2007;25:345–61. 17260393 10.1002/jmri.20808

[71] Trattnig S , Burstein D, Szomolanyi P, Pinker K, Welsch GH, Mamisch TC. T1 (Gd) gives comparable information as Delta T1 relaxation rate in dGEMRIC evaluation of cartilage repair tissue. Invest Radiol 2009;44:598–602. 19692842 10.1097/RLI.0b013e3181b4c236

[72] Bashir A , Gray ML, Burstein D. Gd(DTPA)^2-^ as a measure of cartilage degradation. Magn Reson Med 1996;36:665–73. 8916016 10.1002/mrm.1910360504

[73] Bashir A , Gray ML, Hartke J, Burstein D. Nondestructive imaging of human cartilage glycosaminoglycan concentration by MRI. Magn Reson Med 1999;41:857–65. 10332865 10.1002/(sici)1522-2594(199905)41:5<857::aid-mrm1>3.0.co;2-e

[74] Watanabe A , Wada Y, Obata T, Ueda T, Tamura M, Ikehira H, Moriya H. Delayed gadolinium-enhanced MR to determine glycosaminoglycan concentration in reparative cartilage after autologous chondrocyte implantation: preliminary results. Radiology 2006;239:201–8. 16484349 10.1148/radiol.2383050173

[75] Burstein D , Velyvis J, Scott KT, Stock KW, Kim YJ, Jaramillo D, Boutin RD, Gray ML. Protocol issues for delayed Gd(DTPA)^2-^ enhanced MRI (dGEMRIC) for clinical evaluation of articular cartilage. Magn Reson Med 2001;45:36–41. 11146483 10.1002/1522-2594(200101)45:1<36::aid-mrm1006>3.0.co;2-w

[76] Maroudas A , Muir H, Wingham J. The correlation of fixed negative charge with glycosaminoglycan content of human articular cartilage. Biochim Biophys Acta 1969;177:492–500. 4239606 10.1016/0304-4165(69)90311-0

[77] Fu SX , Cai ZY, Ai H. Stimulus-responsive nanoparticle magnetic resonance imaging contrast agents: design considerations and applications. Adv Healthcare Mater 2021;10:2001091. 10.1002/adhm.202001091 32875751

[78] Kurkijarvi JE , Mattila L, Ojala RO, Vasara AI, Jurvelin JS, Kiviranta I, Nieminen MT. Evaluation of cartilage repair in the distal femur after autologous chondrocyte transplantation using T2 relaxation time and dGEMRIC. Osteoarthr Cartil 2007;15:372–8. 10.1016/j.joca.2006.10.001 17110135

[79] Gillis A , Bashir A, McKeon B, Scheller A, Gray ML, Burstein D. Magnetic resonance imaging of relative glycosaminoglycan distribution in patients with autologous chondrocyte transplants. Invest Radiol 2001;36:743–8. 11753146 10.1097/00004424-200112000-00010

[80] Tiderius CJ , Olsson LE, de Verdier H, Leander P, Ekberg O, Dahlberg L. Gd(DTPA)^2-^-enhanced MRI of femoral knee cartilage: a dose-response study in healthy volunteers. Magn Reson Med 2001;46:1067–71. 11746570 10.1002/mrm.1300

