# Supplementary material for: Quantitatively relating magnetic resonance T1 and T2 to glycosaminoglycan and collagen concentrations mediated by penetrated contrast agents and biomacromolecule-bound water
Source: Regen Biomater. 2023 Apr 11;10:rbad035. doi: 10.1093/rb/rbad035 (PMC10191676; doi:10.1093/rb/rbad035)
Supplement: rbad035_Supplementary_Data [file rbad035_supplementary_data.pdf]

SUPPORTING INFORMATION

Quantitatively relating magnetic resonance T_1 and T_2 to glycosaminoglycan and collagen concentrations mediated by penetrated contrast agents and biomacromolecule-bound water

Jingming Gao[1], Xian Xu[2,*], Xiaoye Yu[1], Ye Fu[1], Hongjie Zhang[1], Siyi Gu[1], Dinglingge Cao[1], Quanyi Guo[3], Liming Xu[4], Jiandong Ding[1,*]

[1]State Key Laboratory of Molecular Engineering of Polymers, Department of Macromolecular Science, Fudan University, Shanghai 200438, China

[2]Department of Radiology, General Hospital of PLA, Beijing 100853, China [3]Institute of Orthopedics, The First Medical Center, Chinese PLA General Hospital, Beijing Key Lab of Regenerative Medicine in Orthopedics, Key Laboratory of Musculoskeletal Trauma and War Injuries of PLA, Beijing 100853, China

[4] National Institutes for Food and Drug Control, Beijing 102629, China.

* Corresponding authors. Emails: jdding1@fudan.edu.cn (J.D. Ding); xuxian_301@163.com (X. Xu)

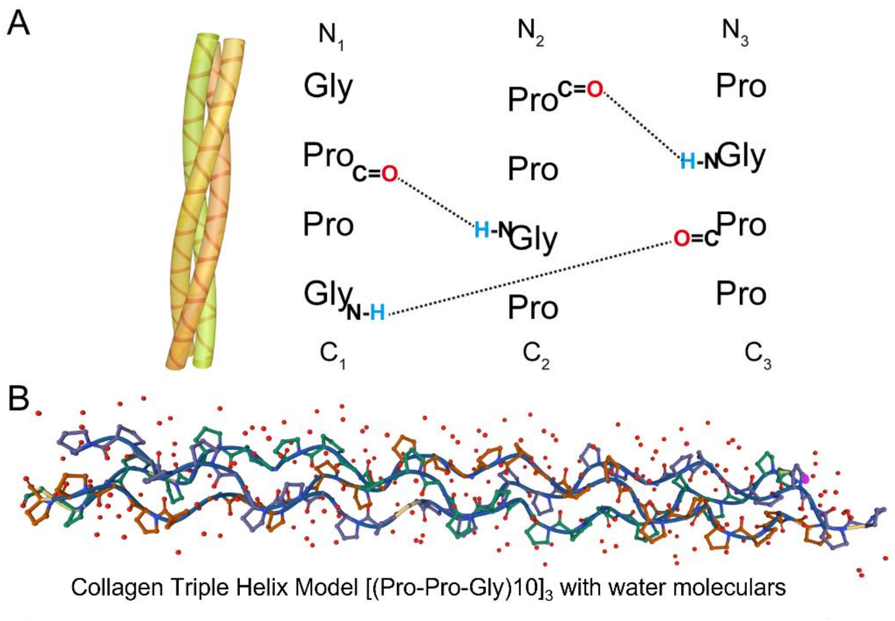

Figure S1. Schematic diagram of collagen structure. (A) The triple helical structure of collagen with hydrogen bonds formed between interchain peptide bonds. (B) Collagen structure (1K6F) from the protein data bank (PDB). The amino and carbonyl groups on collagen combine with surrounding water molecules through hydrogen bonding.

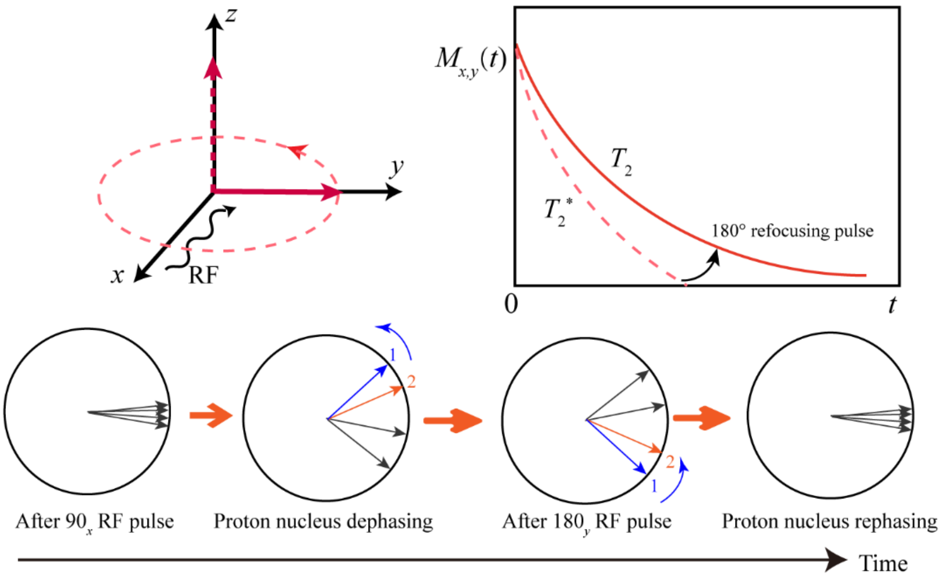

Figure S2 Schematic presentation of the functions of 90° and 180° focusing pulses. The 90° radio frequency (RF) pulse deflects the proton magnetization vector to the *xy*-plane. Due to the inhomogeneity of the main magnetic field, protons with different precession frequencies begin to dephase, and the transverse relaxation vector decays exponentially at a rate determined by exp($-t/T_2*$). A 180° pulse is applied to the *xy*-plane to rephase the proton spins. In our experiment, we set a 180° pulse in the *y* direction to reunite the phase of the proton group.

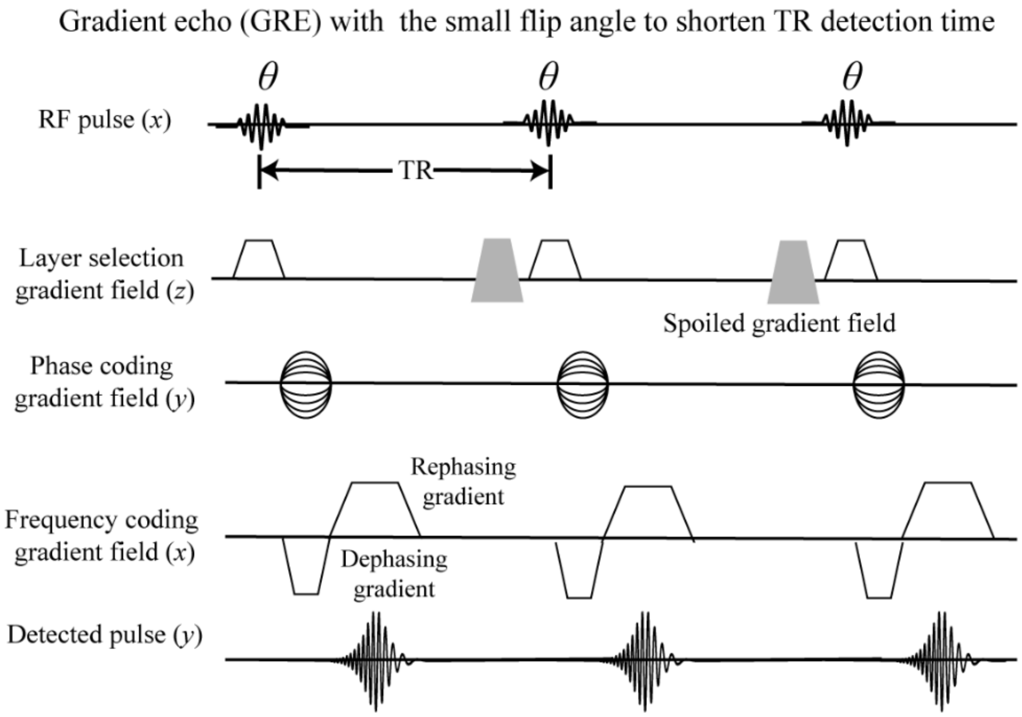

Figure S3 Schematic diagram of gradient echo (GRE) sequence based on small flip angles in the present MRI experiment. The smaller flip angle results in the retention of the larger longitudinal magnetization vectors after the RF pulse, resulting in the recovery of the magnetization vector much faster than that of the 90° pulse. In addition, the conventional 180° rephasing pulse is not used in the GRE sequence, but the nuclear magnetization is dispersed by using the first gradient pulse in the *x* direction, and then another gradient pulse with the opposite polarity is used to form an echo.

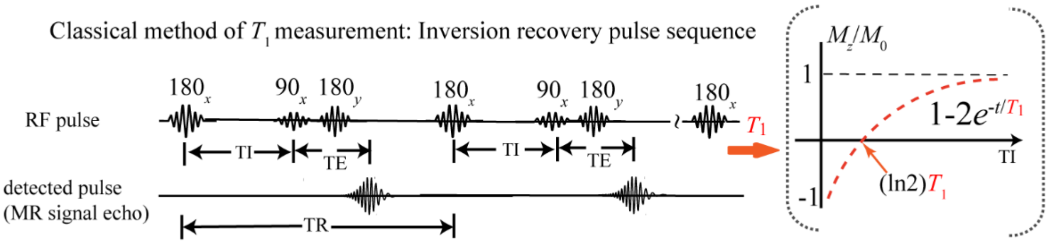

Figure S4 Schematic diagram of the method of the classical T_1 measurement obtained by inversion recovery pulse sequence.

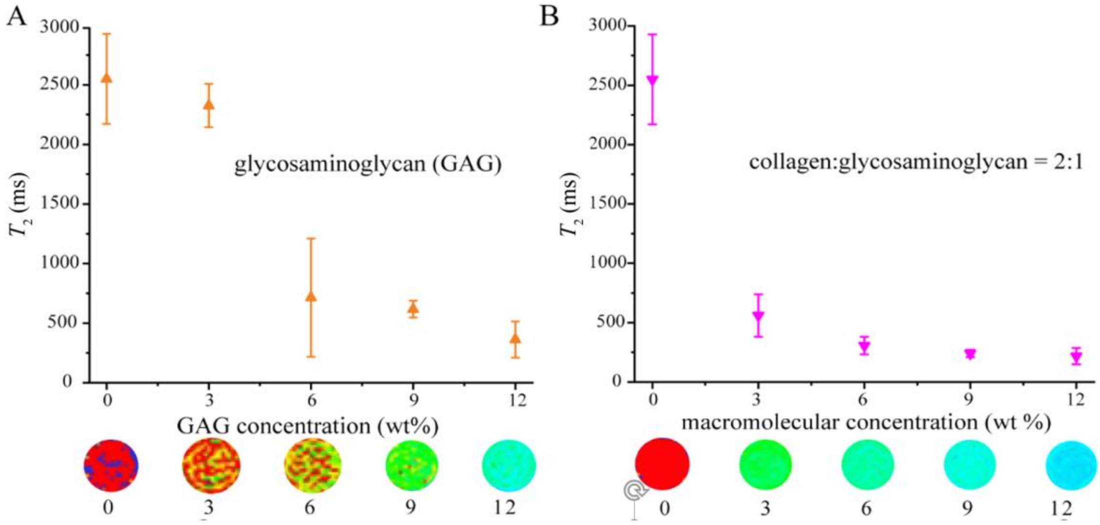

Figure S5. T_2 values as functions of (A) the concentration of glycosaminoglycans (GAG) and (B) that of the mixture of collagen (COL) and GAG. The bottom lines demonstrate some T_2 mapping images. The legend in the upper right indicates the mass ratio between the two biomacromolecules richest in the extracellular matrix of joint cartilage. For each group, n = 3.

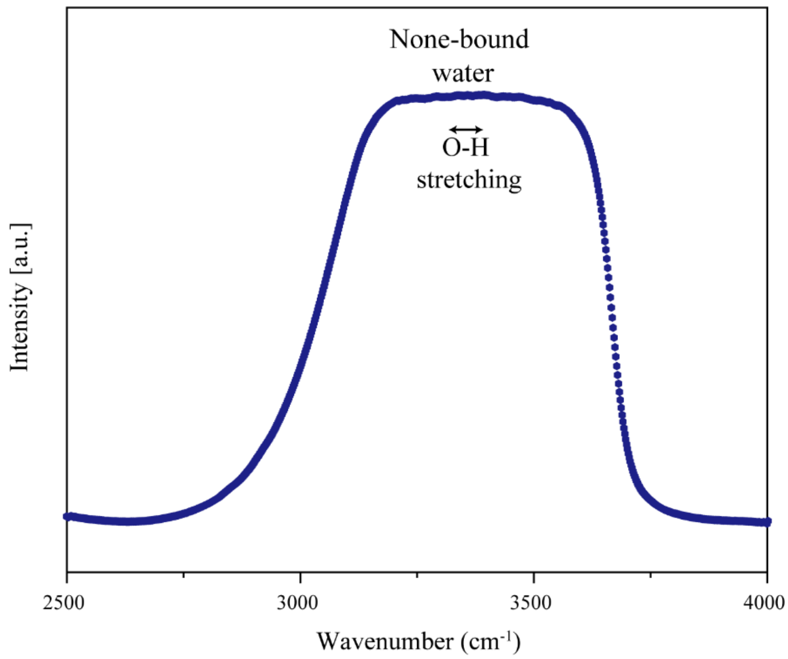

Figure S6. Fourier transform infrared reflectance (FTIR) of pure water without macromolecules. The content of "macromolecular-bound water" in pure water can be considered as zero.

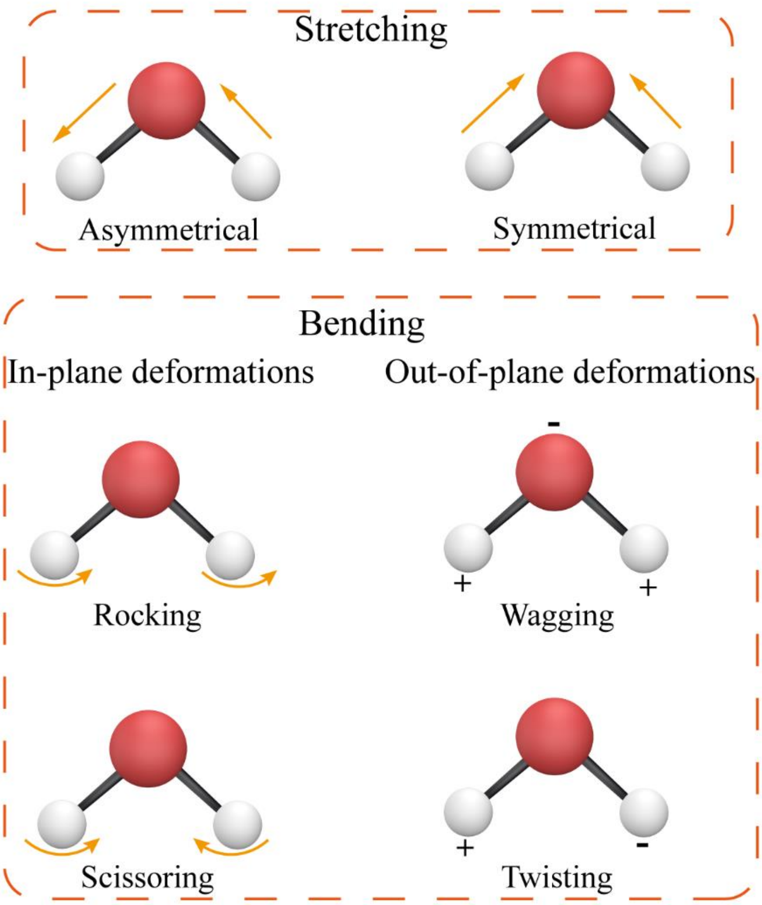

Figure S7. Schematic presentation of classification of IR vibration modes with the two types of stretching vibrations and the four types of bending vibrations.

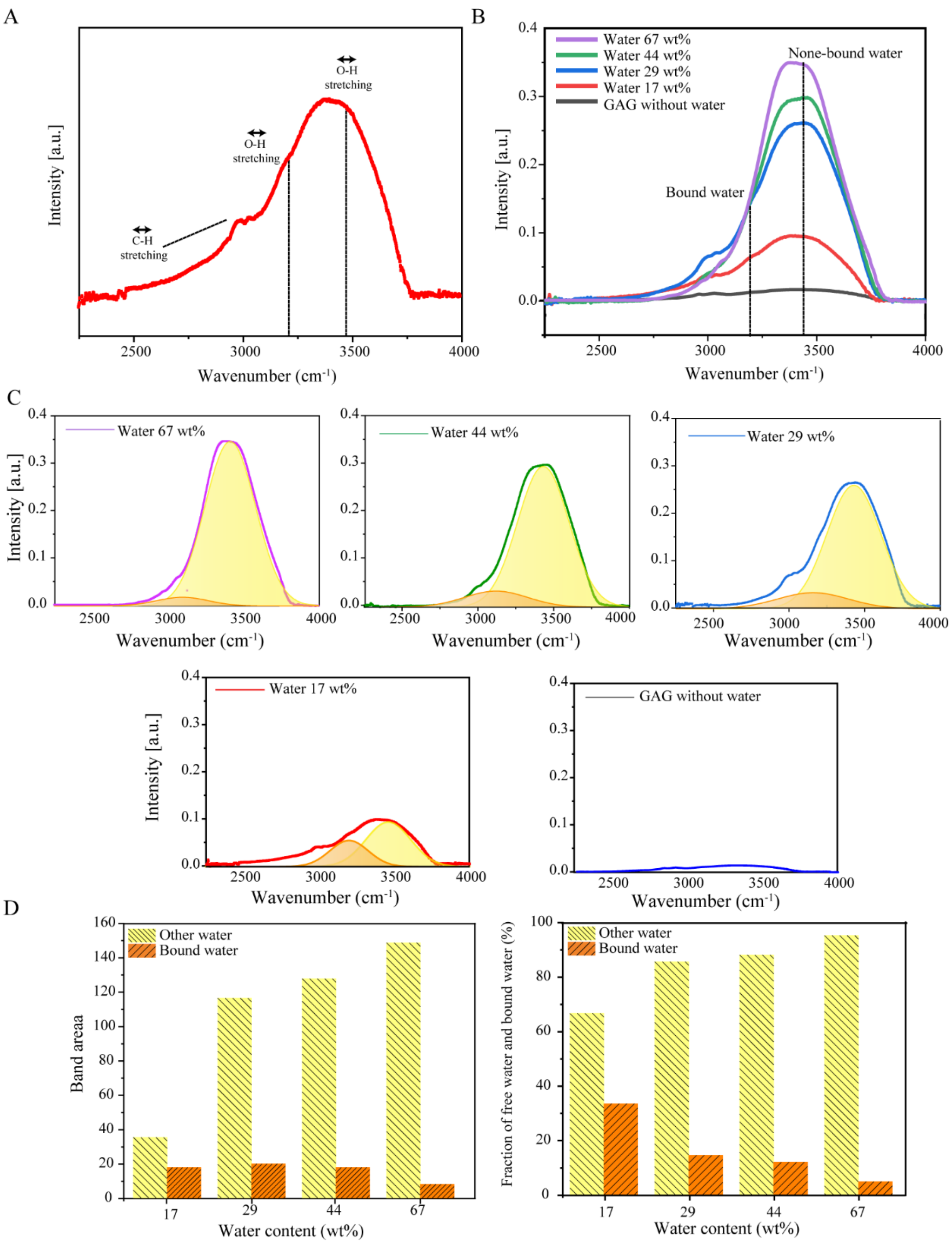

Figure S8. FTIR of the glycosaminoglycan-water system. (A) A demonstrated spectrum with typical absorption peaks corresponding to specific chemical bonds. The formation of hydrogen bonds reduces the force constant of the chemical bond and the absorption frequency shifts to the direction of a low wave number, identified as OH stretching for none-bound water ~3470 cm^{-1}, OH stretching for bound water ~3210 cm^{-1}, CH stretching ~2950 cm^{-1}. (B) Typical FTIR spectra characteristics of the GAG-water system with varied water contents. IR peak positions and their assignments are indicated. (C) Curve fitting of OH stretching bands by Gaussian components. (D) Fitting areas of the different stretching bands and fractions of the indicated water forms as functions of water content.

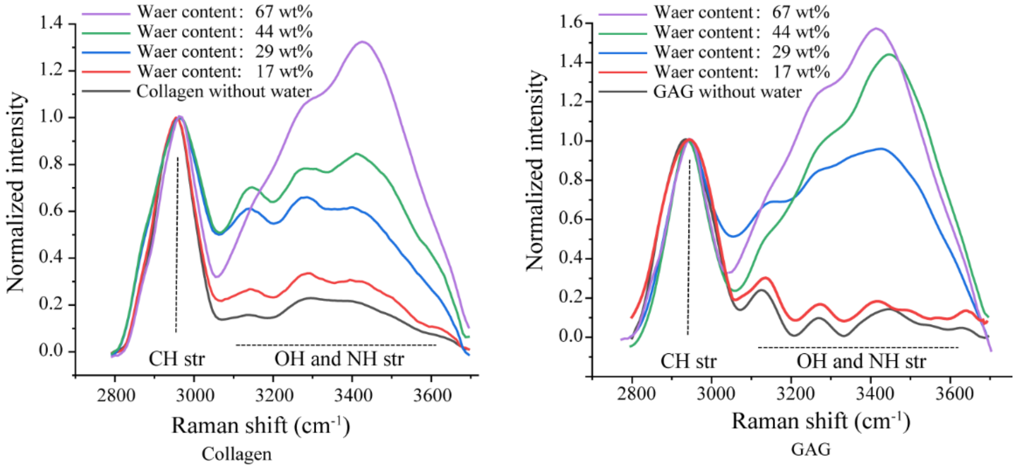

Figure S9. Normalized Raman spectra after baseline correction of the COL-water system and GAG-water system with the indicated different water contents.

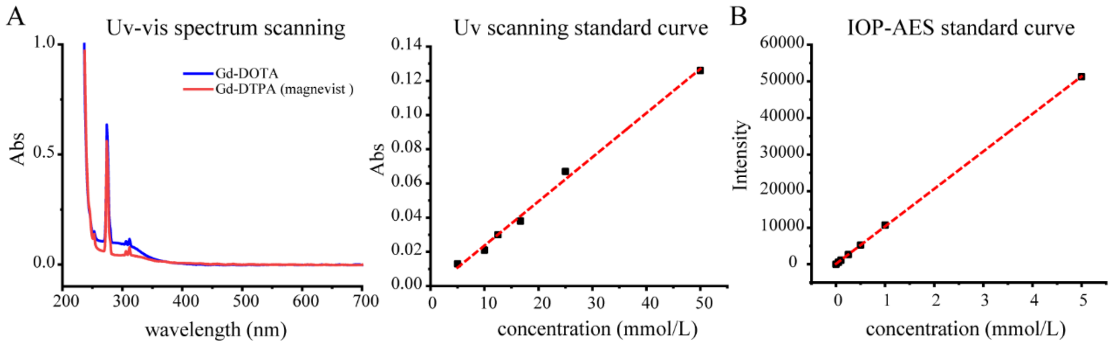

Figure S10. Standard curves for quantitative detection of gadolinium contrast agents. (A) US-visible absorption curves. The gadolinium contrast agent exhibits an obvious absorption peak at 274 nm, and the absorption intensity is proportional to the gadolinium concentration. (B) Standard curve for determination of gadolinium contrast agent content by ICP-AES. This approach can quantify the gadolinium contrast agent with a lower detection limit.
